# Models and Molecular Markers of Spermatogonial Stem Cells in Vertebrates: To Find Models in Nonmammals

**DOI:** 10.1155/2022/4755514

**Published:** 2022-05-31

**Authors:** Hyuk Song, Hyun-Jung Park, Won-Young Lee, Kyung Hoon Lee

**Affiliations:** ^1^Department of Stem Cell and Regenerative Technology, KIT, Konkuk University, Seoul 05029, Republic of Korea; ^2^Department of Animal Biotechnology, College of Life Science and Natural Resources, Sangji University, Wonju-si 26339, Republic of Korea; ^3^Department of Animal Science, Korea National College of Agriculture and Fisheries, Jeonju-si 54874, Republic of Korea

## Abstract

Spermatogonial stem cells (SSCs) are the germline stem cells that are essential for the maintenance of spermatogenesis in the testis. However, it has not been sufficiently understood in amphibians, reptiles, and fish because numerous studies have been focused mainly on mammals. The aim of this review is to discuss scientific ways to elucidate SSC models of nonmammals in the context of the evolution of testicular organization since rodent SSC models. To further understand the SSC models in nonmammals, we point out common markers of an SSC pool (undifferentiated spermatogonia) in various types of testes where the kinetics of the SSC pool appears. This review includes the knowledge of (1) common molecular markers of vertebrate type A spermatogonia including putative SSC markers, (2) localization of the markers on the spermatogonia that have been reported in previous studies, (3) highlighting the most common markers in vertebrates, and (4) suggesting ways of finding SSC models in nonmammals.

## 1. Introduction

**Figure 1:**
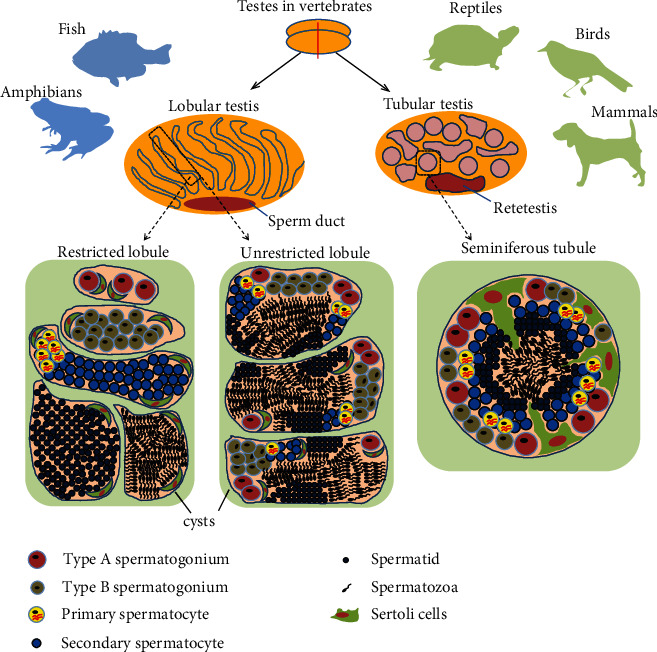
Testicular organization in vertebrates. Amphibians and fish have lobular testes, and the lobular testis is classified into restricted and unrestricted lobules. Type A spermatogonia are found in cysts, located in the periphery of the restricted lobular testis of fish and salamander. Spermiation occurs in the nonperipheral region of the testis. Type A spermatogonia are seen within all cysts which are located in the lobules of the unrestricted lobular testis in fish and frogs. Spermatogenesis occurs in a cyst of a testicular lobule, consisting of unit-termed cysts with a mix of testicular germ cells and Sertoli cells. Mammals, birds, and reptiles have tubular testes where spermatogenesis and type A spermatogonia are observed in the basement membrane of seminiferous tubules. Differentiation of spermatogonia and primary spermatocytes via mitotic cell division and the production of haploid spermatids from the tetraploid primary spermatocytes via meiotic cell division occur in vertebrate spermatogenesis.

The germ cell lineage in both male and female vertebrates originates from primordial germ cells (PGCs). In males, PGCs become enclosed by somatic supporting cells, which are the precursors of Sertoli cells [1, 2]. Sertoli cells of mice and turtles originate from coelomic epithelial cells in the testis; Sertoli and granulosa cells have a common precursor in mice and medaka [1, 3–8]. PGCs and Sertoli cells then together form solid strands of cells, which are called seminiferous cords (or cysts in fish and amphibians) [2, 9]. Later, these cords (or cysts) form a lumen and become lobules in fish and amphibians or seminiferous tubules in reptiles, birds, and mammals [10–12]. Finally, spermatogenesis, which is an organized process in vertebrate testes to produce from spermatogonia (SPG) to mature spermatozoa (SPZ) through an individual's lifespan, occurs in the cyst or seminiferous tubule (Figure 1) [13, 14].

Spermatogonial stem cells (SSCs) are the germline stem cells that are a rare population with long-term renewal potential in the testis [15]. The SSCs are small in proportion, representing only 0.03% of all germ cells in rodent testes because a majority of testicular germ cells are differentiated SPG, spermatocytes (SPC), spermatids (STD), and SPZ in seminiferous tubules or cysts [10, 14, 15]. It has been reported that active movement of SSCs occurs around the vasculature-associated region to communicate with testicular somatic cells [16, 17]. Localization of the SSC pool labeled with Neurogenin 3/enhanced green fluorescent protein (EGFP) is biased to the vascular network and accompanies Leydig and other interstitial cells in the intact testis of mice [17, 18]. The Glial line-derived neurotrophic factor family receptor alpha-1 (GFR*α*-1)+SSC pool tends to localize on the basement membrane of the seminiferous tubules near the vasculature and interstitium [16]. Spermatogonia (SPG) undergoing differentiation leave the vasculature-associated region and disperse throughout the basal compartment of the seminiferous epithelium. The prevailing “A_single_ SSC Model” in seminiferous tubules has been suggested to represent the ability of SSCs to multiply and self-renew in rodents [10, 15, 19–21]. A_single_ (A_s_) SPG as SSCs have the ability to self-renew throughout the lifetime to preserve SSC population size and differentiate into A-_paired_ (A_pr_) and A-_aligned_ (A_al_) SPG to maintain the process of spermatogenesis and preserve male fertility [10, 15, 22, 23]. Currently, there are two rodent SSC models (“revised A_single_ model” and “fragmentation model”), which have been performed with transplantation experiments to characterize stem cell activity in mice [22, 24].

The testicular organization consists of tubular testes for reptiles, birds, and mammals and lobular testes for amphibians and fish. The cysts are produced when a Sertoli cell becomes associated with a primary SPG (also called PGCs), which has the largest nuclei among spermatogenic cells, and mitotic divisions of the primary spermatogonium produce a group of secondary SPG that are enclosed by the Sertoli cell, which forms the wall of the cyst [25, 26]. Spermatogenesis occurs in a cyst of a testicular lobule, which consists of unit-termed cysts, including a mix of testicular germ cells and Sertoli cells [27] (Figure 1). Sperms are released into the lumen of lobules and are transported through sperm ducts connected between each lobule (Figure 1) [28]. Lobular testes are divided into restricted and unrestricted lobular testes, wherein during active spermatogenesis, type A SPG are found only in the periphery of the restricted lobular testis of fish and salamander or type A SPG are seen in all the lobules of the unrestricted lobular testis in fish and frogs, respectively (Figure 1) [25, 28–39]. In the tubular testis, PGCs and pre-Sertoli cells then together form solid strands of cells, which are called seminiferous cords [3, 40, 41]. These cords form a lumen and become seminiferous tubules in reptiles, birds, and mammals [25, 26] (Figure 1). Spermatogenesis developing from SPG to SPZ occurs in seminiferous tubules (Figure 1). Localization of germ cell differentiation is different between anamniote and amniote vertebrates because of diverse testis organization. In addition, the available discussion on the kinetics and models of vertebrate SSC has been limited because identification of SSC models has been reported mainly in mammals. The purpose of this review is to discuss evidence-based SSC models and to find ways for SSC models of nonmammals by searching common SSC and type A SPG markers in vertebrates.

## 2. Models of Spermatogonial Stem Cells and Type A Spermatogonia among Vertebrates

**Figure 2:**
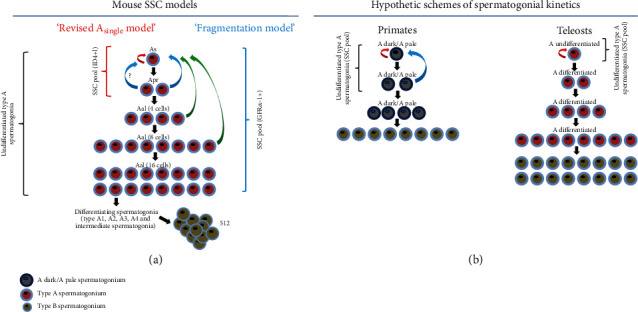
Spermatogonial stem cell models in rodents and proposed kinetics of undifferentiated type A spermatogonia in primates and teleosts. Mouse “revised A_single_ model” and “fragmentation model” are illustrated in (a), based on the previous reports [16, 22, 24, 45, 46]. In vertebrates, only these models have been verified by transplantation experiment which includes SSC markers' expression in undifferentiated type A SPG via GFP transgenic animal model or lineage tracing. ID4 positive A_s_ and A_pr_ SPG are considered the SSC pool in the “revised A_single_ model” (a). In the “fragmentation model,” GFR*α*-1 positive A_s_, A_pr_, and A_al_ SPG are suggested as the SSC pool (a). In addition, duplication of A_s_ SPG by cell division is rare and the majority of A_s_ SPG production occurs by the fragmentation of A_pr_ and A_al_ SPG. Hypothetic schemes of primate and teleost SSCs (undifferentiated SPG) are presented in (b), based on previous reports (b) [23, 42, 73, 75, 116, 117]. However, SSC kinetics has not been verified via transplantation and lineage tracing experiments as performed in mouse SSCs. The SSC pool has been proposed in primates and teleosts; also, the kinetics of the putative SSC pool has not been suggested in birds, reptiles, and amphibians. In primates, A_dark_ and A_pale_ SPG are considered the SSC pool including single and paired A_dark_/A_pale_ spermatogonia (b). Only undifferentiated type A SPG (A undifferentiated) are considered the SSC pool in teleosts (b). Red curved arrows indicate self-renewal of A_s_ spermatogonia (or A_dark_/A_pale_ spermatogonia). Blue curved arrows indicate differentiation from A_pr_ spermatogonia to A_s_ spermatogonia. Green curved arrows indicate clonal fragmentation from A_pr_ and A_al_ spermatogonia to A_s_ spermatogonia. The black arrow indicates a division of each germ cell during spermatogenesis. A_single_, A_pr_, and A_al_ indicate A_s_, A_pr_, and A_al_ spermatogonia. A_dark_/A_pale_ indicates A_dark_/A_pale_ spermatogonia. “A undifferentiated” and “A differentiated” indicate undifferentiated type A and type A differentiated spermatogonia in panel (b), respectively.

The transplantation technique is a powerful tool method to characterize SSC activity including germ cell differentiation, progeny production, and lineage tracing using EGFP-transgenic mice, which has been used for demonstrating mouse SSC models [15, 42]. Currently, there are two models in rodents that have been verified via transplantation with a specific marker. “A_single_ SSC Model” has been modified by Lord and Oatley who subsequently proposed the “revised A_single_ model” (Figure 2(a)) [24]. A previous study has demonstrated the expression and function of inhibitor of differentiation 4 (ID4) in ID4/EGFP mice, wherein the ID4/EGFP-expressing SPG population with high ID4 expression was enriched with SSCs [43]. Spermatogonia with high levels of ID4/EGFP expression are primarily A_s_ SPG within the testes, and these cells encompass over 85% of the SSC population in transplantation analysis. In contrast, SPG with low levels of ID4/EGFP expression are identified primarily as A_pr_ and some A_s_ SPG with low levels of ID4 expression encompass less than 15% of the self-renewing population in transplantation analysis. The SPG population with the high level of ID4/EGFP expression gives higher levels of putative SSC markers, which led to high colonization efficiency in transplantation, and ID4 overexpression impairs spermatogenesis characterized by a blockade in differentiation [43, 44]. They proposed the “revised A_single_ model” which explains that under steady-state conditions, a subset of the A_s_ population with higher ID4 expression is considered functionally true SSCs, and some plasticity of A_s_ and A_pr_ SPG with lower ID4 expression may exist between SSCs and progenitors (Figure 2(a)).

Yoshida and his colleagues proposed the “fragmentation model” which describes cell kinetics and undifferentiated type A SPG in the mice (Figure 2(a)) [16, 22, 45, 46]. The SSC maintenance not only is dependent on self-renewing cells (A_s_) but also involves a more extensive population comprising A_pr_ and A_al_ SPG [47]. This population has self-renewing abilities similar to those of stem cells [47]. Live cell imaging and lineage tracing experiments involving EGFP/GFR*α*-1+SSC pool (including A_s_, A_pr_, and A_al_ SPG) during steady-state spermatogenesis have revealed that the SCC pool actively migrates over a large area on the basal lamina without stopping at particular points and that the breakage of intercellular bridges occurs more often than expected [16]. A_single_ SPG are generated through self-renewal and the fragmentation of A_pr_ or A_al_ SPG [16]. Notably, the production of two A_s_ SPG by cell division is rare, and the majority of A_s_ SPG are generated from the fragmentation of A_pr_ and A_al_ SPG (Figure 2(a)) [16]. This result supports that SSC maintenance is more regulated by the fragmentation of A_pr_ and A_al_ SPG than SSC self-renewal. Recently, they have analyzed the fate of transplanted mouse SSCs at the single-cell resolution that a small fraction of EGFP/GFR*α*-1+SSCs repopulate over the long term in host mouse testes, and it is enhanced to restore host fertility by transient suppression of donor SSC differentiation using retinoic acid [48]. Interestingly, this model is indirectly supported by another study, which reported that purified mouse KIT protooncogene receptor tyrosine kinase+differentiating SPG committed to undergo differentiation can generate functional germinal stem cells that can repopulate germ cell-depleted testes when transplanted into adult mice [49]. This study suggested that stemness could be acquired by differentiating progenitors after tissue injury and throughout life. These findings suggest that the SSC pool is not a fixed entity but a differentiation state that can be lost or regained according to its physical status, thus proposing a new characteristic of the SSC pool [22]. A_single_ and A_pr_ SPG have been suggested as the SSC pool in the “revised A_single_ model”, and the “fragmentation model” suggests that A_s_, A_pr_, and A_al_ SPG are considered the SSC pool (Figure 2(a)). So far, these models are the only evidence-based SSC models in vertebrates.

In primates, dark and pale type A SPG (A_dark_ and A_pale_ SPG) are localized at the basement membrane of primate seminiferous tubules and are morphologically identified by their nuclear architecture and staining intensity with hematoxylin [50, 51]. In the A_dark_ and A_pale_ SPG model, two existing types of SSCs have been suggested. The first type comprises monkey A_dark_ SPG (“reserve” stem cells), which are the stem cells that produce equal numbers of A_dark_ SPG, whereas the second type is the A_pale_ SPG (“active” stem cells), which divide to give rise to type B SPG that differentiate into primary SPC [52–54]. This model has been contested by Ehmcke et al. who claimed that the A_dark_ SPG (“regenerative reserve”) are recognized as true SSCs with a low mitotic activity under steady-state conditions, and the A_pale_ SPG initiate spermatogenesis by self-renewal of A_pale_ SPG as “renewing progenitors” [23, 54–56]. Currently, it has been suggested that the different nuclear architecture of A_dark_ and A_pale_ SPG may strongly correlate with cell cycle stages; A_dark_ and at least some A_pale_ are the same population of cells at different stages of the cell cycle [42, 54, 57]. In terms of the cell cycle, A_dark_ and A_pale_ SPG are considered the SSC pool (undifferentiated type A SPG) (Figure 2(b)). However, the identification of distinct SSCs in the A_dark_ and A_pale_ SPG, which is demonstrated by transplantation experiments with molecular markers, remains unclear.

In birds, the model of SSC identification and renewal has not been established due to insufficient studies on SSCs. The classification of SPG, based on chromatin distribution and nuclear morphology, has been proposed to describe the process of SPG development in birds. In Japanese quail and goose, four different types of SPG are identified in seminiferous tubules: dark type A, two pale A types (pale 1 and 2), and type B SPG [58–60]. In turkey, three types of SPG have been defined: dark type A, pale A type, and type B SPG [61]. The dark type A SPG for both species are analogous to the A_s_/A_pr_ in rodents and A_dark_/A_pale_ in primates.

In reptiles, it has been reported that there are type A and B SPG based on histological morphology. The seasonal cycle of spermatogenesis has demonstrated different patterns with an increase in seminiferous tubule size in turtles, snakes, and lizards [62–66]. In turtles, there are three major types of SPG, namely, resting, type A, and type B SPG [67]. The resting SPG, which appear during four seasons and have darkly stained chromatin packed tightly within the nuclei and lack visible nucleoli, do not enter meiosis to replenish the spermatogonial population near the end of spermatogenesis [67]. Turtle resting SPG may correspond to undifferentiated SPG as the SSC pool. In snakes and lizards, type A SPG are determined by morphology without classifying such as undifferentiated type A SPG [62, 65, 66, 68]. In amphibians, primary SPG that are located in the periphery of the lobules have been considered undifferentiated type A SPG undergoing mitosis, and secondary SPG are similar to type B SPG (differentiated SPG) in rodents [25, 28, 33–35]. In bullfrogs and newts, PGCs claimed in seasonal spermatogenesis are designated as primary SPG [34, 69–71]. However, evidence of resting and primary SPG that supports their SSC potentials has not been found in both species yet.

The majority of testicular structures are lobular types in fish (Figure 1). SSCs have been studied in classification, in vitro culture, and transplantation in fish more than in reptiles and amphibians. Spermatogenesis including type A SPG, type B SPG, and primary SPC has been well studied in fish [14, 32, 72]. An initial cyst is formed with undifferentiated type A SPG, which is considered a stem cell and comprises a few Sertoli cells. Fish type A SPG are classified into undifferentiated type A and differentiated type A SPG that correspond to undifferentiated A_s_~A_al_ (2~16 germ cells) and differentiating type A (A1~intermediate) SPG in rodents, respectively [14, 73–76] (Figure 2(b)). The potential activity of SSCs has been evaluated in fish using transplantation experiments, indicating the stemness of undifferentiated type A SPG. In trout, DEAD-box helicase 4 (VASA)/GFP-expressing PGCs or type A SPG, which are xenotransplanted into salmon, can develop into functional sperm cells and egg cells that can form offspring [77–80]. In addition, the SSC activity of type A SPG has been evaluated using molecular markers [72, 80]. In sturgeon, early-stage germ cells which are transplanted into recipient larvae can migrate to genital ridges and the number of SSCs significantly increases in later larval stages [81]. In zebrafish, VASA/EGFP-expressing undifferentiated type A SPG can develop into spermatogenic cells at different stages of spermatogenesis and oocytes after transplantation into germ cell-deficient male and female zebrafish [82]. Furthermore, in vitro culture and purification of type A SPG have been studied for differentiation and enrichment in dogfish, catfish, zebrafish, and carp [83–88]. So far, undifferentiated type A SPG have the SSC potential in fish (Figure 2(b)).

Undifferentiated type A SPG of vertebrates, such as A_s_, A_pr_, A_al,_ A_dark_, and A_pale_ SPG, are considered the SSC pool, based on their localization in the basement membrane of tubules and cysts of lobules, characterization of nuclei, their rarity, and transplantation evidence. To further understand SSCs in nonmammals, we analyzed the expression of putative SSC markers in type A SPG during the early and adult stages of testis development among vertebrates.

## 3. Class-Crossed Molecular Markers of Spermatogonial Stem Cells and Type A Spermatogonia in Vertebrates

Gonocytes and PGC, which are considered to be the origin of SSCs and also expressed in adult type A SPG, have been used for identifying the SSC pool (Supplementary Table 1) [15]. Putative SSC markers and type A SPG, expressed in testicular tubules (or lobules) and cultured (or isolated) SPG, have been analyzed in the SSC pool of vertebrates; class-crossed and class-specific markers are shown in Supplementary Information including a detailed description of expression of the SSC and SPG markers in vertebrates. According to the “revised A_single_ model” and the “fragmentation model,” the vertebrate SSCs include undifferentiated type A SPG as the SSC pool such as A_s_, A_pr_, and A_al_ SPG or A_dark_ and A_pale_ SPG. “SSCs” claimed in the previous research studies were intactly used in the marker description of this section and Supplementary Information. The candidate markers for the SSC pool, which are common in two classes or more, were selected by results visualized by immunocytochemistry, immunohistochemistry, in situ hybridization, magnetic-activated cell sorting (MACS), and fluorescent-activated cell sorting (FACS) in the testicular tubules (or lobules) at specific developmental stages as well as transplantation experiments for producing donor-derived offspring. SSC-related studies only with RT-PCR results were excluded from the selection of putative SSC markers. All genes listed were confirmed for their evolutionary conservation using NCBI Orthologs (https://www.ncbi.nlm.nih.gov). Due to the number of research reports on nonmammals, it was not easy to isolate the common markers for the SSC pool of vertebrates. As shown in Supplementary Tables 1 and 2, plenty of putative SSC markers from mammalian testis tissues and their cultured (or isolated) SSCs have been studied more frequently than birds, reptiles, amphibians, and fish. Comparatively, GFR*α*-1, thymocyte differentiation antigen 1 (THY1), promyelocytic leukemia zinc finger protein (PLZF), nanos C2HC-type zinc finger 1 (NANO1), nanos C2HC-type zinc finger 2 (NANOS2), and OCT4 are class-crossed markers of the SSC pool (Supplementary Table 1). Here, we review GFR*α*-1 expression in vertebrates which is the most common molecular marker after investigating putative SSC markers.

In mature male dogfish, GFR*α*-1 is highly expressed in all undifferentiated SPG and differentiating SPG, as well as in cultured GFR*α*-1-expressing spermatogonial cells, but it is not detectable in SPC- and STD-related zones [83, 89]. In tilapia, GFR*α*-1 is detected exclusively in undifferentiated type A SPG with a large nucleus of large single cells in sexually mature male testes, and the density of GFR*α*-1+SPG is high in the peripheral regions of the tubular testis (near tunica albuginea). Cultured GFR*α*-1+SPG, isolated from the adult testis, can colonize in recipient adult tilapia [90]. In rainbow trout, GFR*α*-1 transcripts are detected in type A SPG of mature testes, and their levels decrease in type B SPG [91]. In medaka adult testes, GFR*α*-1 transcript levels are high in SPG and moderate in SPC, and SPG isolated from immature testes express GFR*α*-1 [92, 93]. In bullfrogs, PGCs (gonocyte-like SSCs) of adult testes are the largest cells located in the lobular periphery and are surrounded by Sertoli cells, and GFR*α*-1 immunoexpression is observed in the cytoplasm and plasma membrane of PGCs [35]. In adult scorpion mud turtles, GFR*α*-1 is expressed in undifferentiated type A SPG (SSC) and is predominantly located in areas where a seminiferous tubule faces the interstitial compartment containing blood vessels [94]. In chicken, the proportion of GFR*α*-1+ cells is 2.8% in the cells of adult testes. GFR*α*-1 mRNA and protein expression is detected mainly in type A SPG close to the basement membrane of the seminiferous tubule, and GFR*α*-1-expressing SPG produce the progenies in recipient chickens [95, 96]. Spermatogonia, isolated from juvenile and adult quail using a differential plating technique, express GFR*α*-1, and SPG cultured from adult pheasant testes also express GFR*α*-1 [97, 98]. Mammalian GFR*α*-1 is expressed in gonocytes and undifferentiated type A SPG of the testis and cultured (or isolated) SSCs (Supplementary Table 1). Certainly, mouse GFR*α*-1 is expressed in the SSC pool (A_s_, A_pr_, and A_al_ SPG) during steady-state spermatogenesis (Supplementary Table 1). In vertebrates, GFR*α*-1 is a common marker for the SSC pool, and its expression is exclusively observed in gonocytes, undifferentiated type A SPG, and cultured SSCs. In addition, GFR*α*-1+ cells have been used for transplantation experiments to produce donor-derived offspring and for elucidating SSC models in rodents [16, 48]. In analysis of molecular markers, it reveals that GFR*α*-1 is the most potential SSC marker in vertebrates.

## 4. Finding Models of Spermatogonial Stem Cells in Nonmammals

After analyzing type A SPG localization of putative SSC markers in vertebrates, several characteristics are revealed in the SSC pool of vertebrates. Firstly, the SSC pool is observed in SPG in the basement membrane of seminiferous tubules, the periphery of restricted lobular testes, and the basement membrane near the cyst of unrestricted lobular testes mixed with several types of germ cells. Secondly, many of the putative SSC markers in mammals are not expressed or have not been studied in other classes (Supplementary Table 1). Thirdly, transplantation including SSC markers' expression with progeny production has been performed in a few gonochoristic fish (nonmammals). Based on these, we discuss ways of verifying SSC models of nonmammals in the evolution of the testicular organization.

As mentioned above, testis organization is divided into tubular and lobular testes for optimal reproductive strategies. Mammals and birds exhibit homeothermy, internal fertilization, specific sex chromosome (ZZ/ZW or XX/XY), tubular testis, and exogenous factor-independent sex determination (Table 1). Reptiles and amphibians show poikilothermy, external fertilization, lobular testis, and exogenous factor-dependent sex determination; additionally, certain species possess specific sex chromosomes (ZZ/ZW or XX/XY) (Table 1). Fish exhibit poikilothermy, external fertilization, lobular testis, various types of sex chromosomes, and exogenous factor-dependent sex determination (Table 1). Reptilian reproductive strategies show the intermediate characteristics between fish-amphibians and birds-mammals (Table 1). Tubular and lobular testes diverge in stability of the sex determination system, fertilization strategy, and animal body type (Table 1). Although spermatogenesis is a universal process in vertebrates, the testicular organization is varied in the reproductive strategies of vertebrates (Figure 1 and Table 1). The SSC pool of amniotes and anamniotes exists in the basement membrane of seminiferous tubules and cysts of lobular testes, respectively (Figure 1). In particular, the difference in testis organization may imply a difference in SSC kinetics in vertebrates and suggest that the SSC kinetics and their markers can vary between tubular and lobular testes. To understand SSC models in nonmammals further, it is necessary to demonstrate whether the “revised A_single_ models,” “fragmentation model,” or other models can be applied to elucidate SSC kinetics in the lobular testis of anamniotes or not. In a previous study, GFR*α*-1, which has been used to trace mouse A_s_, A_pr_, and A_al_ SPG to explain the “fragmentation model,” was found to be the only common marker for the vertebrate SSC pool (Supplementary Table 1) [16, 48]. Comparatively, type A SPG of fish are classified in more detail than those of reptiles and birds (Figure 2(b)). In addition, the kinetics of undifferentiated type A SPG using transgenic fish and organ culture has been reported in several studies. In rainbow trout and medaka, VASA/GFP-carrying type A SPG have been used in transplantation experiments and germ cell cultures, which possess the ability to produce donor-derived offspring [77–80, 99–109]. Additionally, the culture of testis fragments has been performed in rainbow trout and medaka, which increased the proliferation of SPC and SPG; GFR*α*-1 is expressed in undifferentiated type A SPG of both species [110, 111] (Supplementary Table 1). Recently, the in vitro-expanded germline stem cells, enriched from immature VASA/GFP rainbow trout, exhibit stem cell activity and potency to produce functional eggs, sperm, and healthy offspring [109]. In testicular organization, medaka and rainbow trout have restricted and unrestricted lobular testes, respectively [79, 91, 110, 112–114]. In common, two bony fish are gonochoristic in which only each individual develops to a male with testes or a female with ovaries after fertilization [115]. Spermatogenesis in unrestricted lobular testes occurs in a single cyst corresponding to the seminiferous tubules (Figure 1). However, the differentiation and proliferation of germ cells (SPG, SPC, STD, and SPZ) occur in various cysts of restricted lobular testes, and the cysts are located in the peripheral region extending to the central sperm duct; each cyst in the testis is divided into mitotic and meiotic cysts in restricted lobular testes (Figure 1). It is possible that SSC kinetics (self-renewal and division) is different from those in unrestricted lobular and tubular testes. Rainbow trout and medaka satisfy the conditions (GFR*α*-1 expression in undifferentiated SPG, organ culture of the testis, and establishment of a GFP transgenic animal model) that can apply the live imaging experiments using GFR*α*-1/EGFP to prove the “fragmentation model”; SCC activity can be analyzed in ID4-EGFP salmon and medaka to verify the “revised A_single_ model.” In addition, PLZF and OCT4, which are conserved in vertebrates, are promising undifferentiated SPG markers after GFR*α*-1. Therefore, finding SSC models using two fish can provide the diversity of SSC kinetics in the lobular testis in nonmammals.

Here, we tried to investigate the common SSC markers after examining the putative markers of the SSC pool in vertebrates. To date, GFR*α*-1 is the most common marker for the SSC pool in vertebrates, and it can be used to verify SSC models using experiments (transgenic animal model, transplantation, and in vitro culture) in nonmammals. To understand SSC kinetics in nonmammals, further studies including the identification of novel markers, development of organ culture, and establishment of SSC molecular marker-carrying transgenic animal models should be performed in birds, reptiles, and amphibians. If a specific SSC model is common across several classes or a new model of SSCs can appear in other classes with lobular testes, we can understand why self-renewal and differentiation of SSCs are different in the evolution of testis organization and reproductive strategy among vertebrates.

## Figures and Tables

**Table 1:** Reproductive strategies in vertebrates.

Reproductive strategy	Subgroups				
	Fish	Amphibians	Reptiles	Birds	Mammals
Animal body types	Poikilothermic	Poikilothermic	Poikilothermic	Homeothermic	Homeothermic
Fertilization	External^∗^	External^∗^	Internal	Internal	Internal
Sex chromosome types	XX/XY, ZZ/ZW, X1X2X3X4/X1X2Y, ZO/ZZ, or more types [115, 118]	XX/XY or ZZ/ZW [118, 119]	XX/XY or ZZ/ZW [118, 120–123]	ZZ/ZW [118, 124]	XX/XY [118, 125, 126]
Sex reversal or biased sex ratio	Yes [115, 127]	Yes [128–131]	Yes [120, 122, 123, 132]	No	No
Testicular organization	Anastomosing tubular testis, restricted lobular testis, or unrestricted lobular testis [14, 29–32, 115, 133, 134]	Restricted lobular testis or unrestricted lobular testis [25, 28, 33–38]	Tubular testis [62, 65]	Tubular testis [135]	Tubular testis [14, 136–138]

^∗^There are exceptions in fish and amphibians. Guppies, coelacanths, dogfish, and fanged frogs have the internal fertilization [139–143].

## Data Availability

Data sharing is not applicable because no new data was created in this review..

## Supplementary Materials

Supplementary Materials Supplementary Table 1. Class-crossed markers on the spermatogonial stem cell pool and type A spermatogonia in testes and cultured (or isolated) spermatogonia in vertebrates. Supplementary Table 2. Molecular markers on the spermatogonial stem cell pool and type A spermatogonia in testes and cultured (or isolated) spermatogonia in mammals.
