# Supplementary material for: Models and Molecular Markers of Spermatogonial Stem Cells in Vertebrates: To Find Models in Nonmammals
Source: Stem Cells Int. 2022 May 31;2022:4755514. doi: 10.1155/2022/4755514 (PMC9174007; doi:10.1155/2022/4755514)
Supplement: Supplementary Materials — Supplementary Table 1. Class-crossed markers on the spermatogonial stem cell pool and type A spermatogonia in testes and cultured (or isolated) spermatogonia in vertebrates. Supplementary Table 2. Molecular markers on the spermatogonial stem cell pool and type A spermatogonia in testes and cultured (or isolated) spermatogonia in mammals. [file 4755514.f1.docx]

**Supplementary Materials**

**Supplementary Table 1, Supplementary Table 2, and References.**

Models and molecular markers of spermatogonial stem cells in vertebrates: to find models in non-mammals

Hyuk Song^1^, Hyun-Jung Park^2^, Won-Young Lee^3^ and Kyung Hoon Lee^1*^

^1^Department of Stem Cell and Regenerative Technology, KIT, Konkuk University, Seoul, 05029, Republic of Korea.

^2^Department of Animal biotechnology, College of Life Science and Natural Resources, Sangji University, Wonju-si, 26339, Republic of Korea.

^3^Department Department of Animal Science, Korea National College of Agriculture and Fisheries, Jeonju-si 54874, Republic of Korea.

***Correspondence:** Kyung Hoon Lee Ph. D., Department of Stem Cell and Regenerative Technology, KIT, Konkuk University, 120 Neungdongro, Gwangjin-gu, Seoul, 05029, Republic of Korea. Email: djslam@kku.ac.kr, Tel: +82-2-450-0562.

**Supplementary Table 1.** Class-crossed markers on the spermatogonial stem cell pool and type A spermatogonia in testes and cultured (or isolated) spermatogonia in vertebrates.

| **Subgroups**  **Genes** | **Fish** | | **Amphibians** | | **Reptiles** | | **Birds** | | **Mammals** | |
| --- | --- | --- | --- | --- | --- | --- | --- | --- | --- | --- |
|  | **Testis tissue** | **In vitro** | **Testis tissue** | **In vitro** | **Testis tissue** | **In vitro** | **Testis tissue** | **In vitro** | **Testis tissue** | **In vitro** |
| **GFRα-1** | Dogfish [[1](#_ENREF_1), [2](#_ENREF_2)]  Nile tilapia [[3](#_ENREF_3)]  Rainbow trout [[4](#_ENREF_4)]  Medaka [[5](#_ENREF_5)] | Dogfish [[1](#_ENREF_1)]  Nile tilapia [[3](#_ENREF_3)]  Rainbow trout [[4](#_ENREF_4), [6](#_ENREF_6)] | Bullfrog [[7](#_ENREF_7)] |  | Scorpion mud turtle [[8](#_ENREF_8)] |  | Chicken [[9](#_ENREF_9), [10](#_ENREF_10)] | Chicken [[9](#_ENREF_9)]  Quail [[11](#_ENREF_11)]  Pheasant [[12](#_ENREF_12)] | Bull [[13-15](#_ENREF_13)]  Cat [[16](#_ENREF_16), [17](#_ENREF_17)]  Dog [[18](#_ENREF_18)]  Donkey [[19](#_ENREF_19)]  Goat [[20](#_ENREF_20)]  Horse [[19](#_ENREF_19), [21](#_ENREF_21)]  Human [[22-25](#_ENREF_22)]  Monkey [[26-30](#_ENREF_26)]  Mouse [[24](#_ENREF_24), [31-55](#_ENREF_31)]  Mule [[19](#_ENREF_19)]  Peccary [[56](#_ENREF_56), [57](#_ENREF_57)]  Pig [[58-60](#_ENREF_58)]  Rat [[61](#_ENREF_61), [62](#_ENREF_62)] | Bull [[13-15](#_ENREF_13), [63-65](#_ENREF_63)]  Cat [[16](#_ENREF_16)]  Dog [[18](#_ENREF_18)]  Goat [[20](#_ENREF_20), [66](#_ENREF_66), [67](#_ENREF_67)]  Human [[22](#_ENREF_22), [68-73](#_ENREF_68)]  Horse [[21](#_ENREF_21)]  Monkey [[30](#_ENREF_30)]  Mouse [[39](#_ENREF_39), [41](#_ENREF_41), [54](#_ENREF_54), [74-82](#_ENREF_74)]  Pig [[58-60](#_ENREF_58), [83-86](#_ENREF_83)]  Rabbit [[87](#_ENREF_87)]  Rat [[61](#_ENREF_61), [62](#_ENREF_62)] |
| **INTEGRIN- α6** **(CD49f)** |  |  |  |  |  |  |  | Chicken [[88-98](#_ENREF_88)]  Quail [[11](#_ENREF_11)] | Goat [[20](#_ENREF_20)]  Human [[23](#_ENREF_23), [99](#_ENREF_99)]  Monkey [[29](#_ENREF_29)] | Bull [[100](#_ENREF_100)]  Goat [[20](#_ENREF_20), [67](#_ENREF_67), [101-104](#_ENREF_101)]  Human [[22](#_ENREF_22), [99](#_ENREF_99), [105](#_ENREF_105), [106](#_ENREF_106)]  Mouse [[39](#_ENREF_39), [107-111](#_ENREF_107)]  Monkey [[29](#_ENREF_29)]  Pig [[112](#_ENREF_112)] |
| **INTEGRIN-ß1 (CD29)** |  |  |  |  |  |  |  | Chicken [[89](#_ENREF_89), [91](#_ENREF_91), [92](#_ENREF_92), [94](#_ENREF_94), [96-98](#_ENREF_96), [113](#_ENREF_113)] |  | Bull [[100](#_ENREF_100)]  Goat [[101](#_ENREF_101), [103](#_ENREF_103), [104](#_ENREF_104)]  Mouse [[82](#_ENREF_82), [107](#_ENREF_107), [108](#_ENREF_108), [111](#_ENREF_111)]  Pig [[112](#_ENREF_112), [114](#_ENREF_114)] |
| **THY1 (CD90)** | Rohu [[115](#_ENREF_115)] | Catfish [[116](#_ENREF_116)] |  |  |  |  | Chicken [[117](#_ENREF_117)] |  | Buffalo [[118](#_ENREF_118)]  Bull [[119](#_ENREF_119)]  Goat [[120](#_ENREF_120)]  Mouse [[39](#_ENREF_39)]  Human [[99](#_ENREF_99), [121](#_ENREF_121), [122](#_ENREF_122)]  Treeshrew [[123](#_ENREF_123)] | Buffalo [[118](#_ENREF_118)]  Bull [[13](#_ENREF_13), [100](#_ENREF_100)]  Goat [[101](#_ENREF_101), [103](#_ENREF_103), [120](#_ENREF_120), [124](#_ENREF_124)]  Human [[22](#_ENREF_22), [73](#_ENREF_73), [99](#_ENREF_99)]  Monkey [[27](#_ENREF_27), [29](#_ENREF_29), [30](#_ENREF_30), [125](#_ENREF_125)]  Mouse [[39](#_ENREF_39), [126](#_ENREF_126), [127](#_ENREF_127)]  Pig [[112](#_ENREF_112), [128](#_ENREF_128)]  Treeshrew [[123](#_ENREF_123)] |
| **SSEA-1 (CD15)** | Rohu [[115](#_ENREF_115)] |  |  |  |  |  |  | Chicken [[97](#_ENREF_97), [129-133](#_ENREF_129)] | Blackbuck [[134](#_ENREF_134)]  Cat [[135](#_ENREF_135)]  Mouse [[136](#_ENREF_136)]  Rat [[137](#_ENREF_137)]  Tammar [[138](#_ENREF_138)] | Cat [[135](#_ENREF_135)]  Pig [[112](#_ENREF_112), [139](#_ENREF_139)]  Mouse [[39](#_ENREF_39)] |
| **SSEA-4** |  |  |  |  |  |  |  | Chicken [[133](#_ENREF_133)] | Cat [[135](#_ENREF_135)]  Monkey [[29](#_ENREF_29), [140](#_ENREF_140)]  Human [[99](#_ENREF_99), [121](#_ENREF_121), [122](#_ENREF_122), [141](#_ENREF_141)] | Cat [[135](#_ENREF_135)]  Human [[68](#_ENREF_68), [99](#_ENREF_99), [106](#_ENREF_106), [141](#_ENREF_141), [142](#_ENREF_142)]  Monkey [[29](#_ENREF_29)] |
| **PLZF**  (ZBTB16) | Rohu [[143](#_ENREF_143)]  Catfish [[144](#_ENREF_144)]  Dogfish [[1](#_ENREF_1)]  Rainbow trout [[145](#_ENREF_145)]  Zebrafish [[146](#_ENREF_146)] |  |  |  |  |  |  |  | Bull [[119](#_ENREF_119), [147](#_ENREF_147), [148](#_ENREF_148)]  Cat [[149](#_ENREF_149)]  Dog [[18](#_ENREF_18), [150](#_ENREF_150)]  Donkey [[19](#_ENREF_19)]  Goat [[67](#_ENREF_67), [151](#_ENREF_151)]  Human [[152](#_ENREF_152)]  Horse [[19](#_ENREF_19)]  Mouse [[24](#_ENREF_24), [31-34](#_ENREF_31), [36](#_ENREF_36), [38](#_ENREF_38), [41](#_ENREF_41), [44](#_ENREF_44), [45](#_ENREF_45), [48](#_ENREF_48), [50-54](#_ENREF_50), [82](#_ENREF_82), [126](#_ENREF_126), [127](#_ENREF_127), [153-175](#_ENREF_153)]  Mule [[19](#_ENREF_19)]  Monkey [[26](#_ENREF_26), [27](#_ENREF_27), [30](#_ENREF_30)]  Pig [[86](#_ENREF_86), [176-178](#_ENREF_176)]  Rat [[179](#_ENREF_179)]  Sheep [[180](#_ENREF_180)]  Yak [[181](#_ENREF_181)] | Bull [[13](#_ENREF_13), [119](#_ENREF_119), [147](#_ENREF_147), [148](#_ENREF_148)]  Goat [[67](#_ENREF_67), [101](#_ENREF_101), [102](#_ENREF_102), [104](#_ENREF_104), [120](#_ENREF_120), [124](#_ENREF_124), [151](#_ENREF_151)]  Human [[22](#_ENREF_22), [69](#_ENREF_69), [73](#_ENREF_73), [105](#_ENREF_105), [152](#_ENREF_152), [182](#_ENREF_182)]  Monkey [[30](#_ENREF_30)]  Mouse [[41](#_ENREF_41), [74](#_ENREF_74), [75](#_ENREF_75), [79](#_ENREF_79), [80](#_ENREF_80), [82](#_ENREF_82), [154](#_ENREF_154), [156-159](#_ENREF_156), [162](#_ENREF_162), [172](#_ENREF_172), [183-187](#_ENREF_183)]  Pig [[85](#_ENREF_85), [86](#_ENREF_86), [112](#_ENREF_112), [128](#_ENREF_128), [176](#_ENREF_176), [177](#_ENREF_177), [188](#_ENREF_188)]  Rabbit [[87](#_ENREF_87)]  Rat [[179](#_ENREF_179), [189](#_ENREF_189), [190](#_ENREF_190)]  Sheep [[191](#_ENREF_191), [192](#_ENREF_192)]  Treeshrew [[123](#_ENREF_123)] |
| **NANOS**  **genes** | Flounder [[193](#_ENREF_193)]  Medaka [[194](#_ENREF_194)]  Nile tilapia [[3](#_ENREF_3)]  Rainbow trout [[145](#_ENREF_145)]  Zebrafish [[195](#_ENREF_195)] |  |  |  |  |  |  |  | Goat [[196](#_ENREF_196)]  Horse [[21](#_ENREF_21)]  Human [[197](#_ENREF_197)]  Marmoset [[197](#_ENREF_197)]  Mouse [[34](#_ENREF_34), [36](#_ENREF_36), [49](#_ENREF_49), [50](#_ENREF_50), [198](#_ENREF_198)]  Peccary [[57](#_ENREF_57)]  Rat [[137](#_ENREF_137)] | Goat [[67](#_ENREF_67), [196](#_ENREF_196)]  Horse [[21](#_ENREF_21)]  Rat [[137](#_ENREF_137)] |
| **OCT4 (POU5F1)** | Catfish [[144](#_ENREF_144)]  Dogfish [[2](#_ENREF_2)]  Medaka [[194](#_ENREF_194)]  Rohu [[115](#_ENREF_115)] |  |  |  |  |  |  | Chicken [[199](#_ENREF_199)] | Buffalo [[118](#_ENREF_118), [200](#_ENREF_200), [201](#_ENREF_201)]  Cat [[202](#_ENREF_202)]  Cheetah [[202](#_ENREF_202)]  Human [[99](#_ENREF_99), [122](#_ENREF_122), [197](#_ENREF_197), [203](#_ENREF_203), [204](#_ENREF_204)]  Leopard [[202](#_ENREF_202)]  Marmoset [[197](#_ENREF_197), [205](#_ENREF_205)]  Mouse [[126](#_ENREF_126), [168](#_ENREF_168)]  Pig [[114](#_ENREF_114)]  Rat [[197](#_ENREF_197), [206](#_ENREF_206)] | Buffalo [[118](#_ENREF_118), [200](#_ENREF_200), [201](#_ENREF_201)]  Bull [[63](#_ENREF_63), [100](#_ENREF_100)]  Goat [[66](#_ENREF_66), [67](#_ENREF_67), [103](#_ENREF_103), [104](#_ENREF_104), [124](#_ENREF_124)]  Human [[106](#_ENREF_106), [142](#_ENREF_142)]  Mouse [[75](#_ENREF_75), [154](#_ENREF_154), [163](#_ENREF_163), [172](#_ENREF_172), [183](#_ENREF_183), [185-187](#_ENREF_185)]  Pig [[114](#_ENREF_114), [128](#_ENREF_128)]  Rabbit [[87](#_ENREF_87)]  Sheep [[191](#_ENREF_191)]  Treeshrew[[123](#_ENREF_123)] |
| **DAZL** | Medaka[[207](#_ENREF_207)] |  |  |  |  |  | Chicken [[208](#_ENREF_208), [209](#_ENREF_209)] | Chicken [[91](#_ENREF_91), [130](#_ENREF_130)]  Quail [[11](#_ENREF_11)] | Dog [[18](#_ENREF_18), [150](#_ENREF_150), [210](#_ENREF_210)]  Horse [[211](#_ENREF_211), [212](#_ENREF_212)]  Human [[213](#_ENREF_213)]  Mouse [[42](#_ENREF_42), [214](#_ENREF_214)]  Rat [[206](#_ENREF_206)] | Dog [[150](#_ENREF_150)]  Human [[106](#_ENREF_106)]  Mouse [[76](#_ENREF_76), [157](#_ENREF_157), [186](#_ENREF_186), [187](#_ENREF_187), [214](#_ENREF_214)]  Rat[[190](#_ENREF_190)] |
| **GPR125** |  |  |  |  |  |  |  | Chicken [[199](#_ENREF_199)] | Mouse [[186](#_ENREF_186)] | Cat [[135](#_ENREF_135)]  Human [[22](#_ENREF_22), [70](#_ENREF_70), [71](#_ENREF_71), [73](#_ENREF_73), [141](#_ENREF_141), [182](#_ENREF_182)]  Mouse[[186](#_ENREF_186)] |
| **DND** | Bluefin tuna [[215](#_ENREF_215)]  Medaka [[216](#_ENREF_216)]  Turbot [[217](#_ENREF_217)] | Bluefin tuna [[215](#_ENREF_215)]  Medaka [[216](#_ENREF_216)] |  |  |  |  | Chicken [[208](#_ENREF_208), [218](#_ENREF_218)] |  | Mouse [[34](#_ENREF_34)] |  |
| **Nanog** | Medaka [[219](#_ENREF_219)] |  |  |  |  |  |  |  | Blackbuck [[134](#_ENREF_134)]  Human [[99](#_ENREF_99)]  Monkey [[29](#_ENREF_29)]  Mouse [[220](#_ENREF_220)] | Bull [[63](#_ENREF_63)]  Goat [[67](#_ENREF_67), [124](#_ENREF_124)] |
| **CSF1R** |  |  |  |  | Scorpion mud turtle [[8](#_ENREF_8)] |  |  |  | Donkey [[19](#_ENREF_19)]  Horse [[19](#_ENREF_19)]  Mule [[19](#_ENREF_19)] |  |
| **TRA-1-81**  **(PCLP-1)** |  | Rohu [[115](#_ENREF_115)] |  |  |  |  |  |  | Cat [[135](#_ENREF_135)]  Monkey [[140](#_ENREF_140)] | Cat [[135](#_ENREF_135)] |

*Expression of markers in testis section and cultured (or isolated) spermatogonia is separated in this table.

**1. GFRα-1**

In mature male dogfish, Glial cell line-derived neurotrophic factor family receptor alpha-1 (GFRα-1) is highly expressed in all undifferentiated SPG and differentiating SPG as well as cultured GFRα-1-expressing spermatogonial cells, but it is undetectable in SPC- and STD-related zones [[1](#_ENREF_1), [2](#_ENREF_2)]. In tilapia, GFRα-1 is detected exclusively in type A undifferentiated SPG with a large nucleus of large single cells in sexually mature male testes, and the density of GFRα-1+ SPG is high in the peripheral regions of the tubular testis (near tunica albuginea). Cultured SPG (GFRα-1 positive), isolated from adult testis, can colonize recipient adult tilapia [[3](#_ENREF_3)]. In rainbow trout, GFRα-1 transcripts are detected in type A SPG of mature testes, and their levels decrease in type B SPG [[4](#_ENREF_4)]. In medaka adult testes, GFRα-1 transcript levels are high in SPG and moderate in SPC, and SPG isolated from immature testes express GFRα-1 [[5](#_ENREF_5), [6](#_ENREF_6)]. In bullfrogs, PGCs (gonocyte-like SSC) of adult testes are the largest cells located in the lobular periphery and are surrounded by Sertoli cells, and GFRα-1 immunoexpression is observed in the cytoplasm and plasma membrane of PGCs [[7](#_ENREF_7)]. In adult scorpion mud turtles, GFRα-1 is expressed in type A undifferentiated SPG (SSC pool) and is predominantly located in areas where a seminiferous tubule faces the interstitial compartment containing blood vessels [[8](#_ENREF_8)]. This result is consistent with previous findings obtained in studies on mice [[55](#_ENREF_55), [221](#_ENREF_221)]. In chicken, the proportion of GFRα-1+ cells is 2.8% in the cells of adult testes. GFRα-1 mRNA and protein expression are detected mainly in type A SPG close to the basement membrane of the seminiferous tubule, and GFRα-1-expressing SPG produce the progenies in recipient chickens [[9](#_ENREF_9), [10](#_ENREF_10)]. SPG, isolated from juvenile and adult quail using differential plating technique, express GFRα-1, and SPG cultured from adult pheasant testes also express GFRα-1 [[11](#_ENREF_11), [12](#_ENREF_12)]. In bulls, GFRα-1 is expressed in the SPG of seminiferous tubules of neonatal and prepubertal testes as well as SSC (or undifferentiated SPG), which are cultured and isolated from neonatal, prepubertal, and adult testes, [[13-15](#_ENREF_13), [63-65](#_ENREF_63)]. In cats, GFRα-1 is detected in a single spermatogonium of the basement membrane of seminiferous tubules and SSC colonies cultured from pubertal testes [[16](#_ENREF_16), [17](#_ENREF_17)]. In dogs, GFRα-1 is localized in the cell membrane in the majority of undifferentiated SPG of prepubertal testes, and GFRα-1 is expressed in type A SPG of the basement membrane of seminiferous tubules in adult testes [[18](#_ENREF_18)]. In horses, donkeys, and mules, the expression of GFRα-1 is observed in the nucleus of type A undifferentiated SPG, and SPG isolated from frozen adult horse testes express GFRα-1 [[19](#_ENREF_19), [21](#_ENREF_21)]. In goats, GFRα-1 is present on the plasma membrane of SPG in prepubertal testes, SSC cultured from neonatal testis, and PLZF/EGFP-carrying SSC express GFRα-1 [[20](#_ENREF_20), [66](#_ENREF_66), [67](#_ENREF_67)]. In humans, GFRα-1 is observed in undifferentiated SPG and SSC cultured (or isolated) from adult testes that express GFRα-1 [[22-25](#_ENREF_22), [68-73](#_ENREF_68)]. In monkeys, GFRα-1 is detected in the gonocytes of juvenile testes and undifferentiated SPG of prepubertal and adult testes, including cultured SSC [[26-30](#_ENREF_26)]. In pigs, GFRα-1 protein is detected in the gonocytes of neonatal testis and undifferentiated SPG of prepubertal and adult testes as well as SSC, which were cultured and isolated from neonatal testes, express GFRα-1 and then differentiate into STD. In addition, GFRα-1+ SPG or gonocytes have been used for long-term SSC culture and tracing of germ cell-derived colonies [[58-60](#_ENREF_58), [83-86](#_ENREF_83)]. In rabbits, SPG-derived colonies cultured from prepubertal testes express GFRα-1 [[87](#_ENREF_87)]. In rats, GFRα-1 is expressed in the gonocytes of postnatal testes and a small population of undifferentiated SPG of prepubertal testes, and undifferentiated SPG cultured from prepubertal testes express GFRα-1 [[61](#_ENREF_61), [222](#_ENREF_222)]. In mice, GFRα-1 is expressed in A_s_, A_pr_, and A_al_ undifferentiated SPG of adult testes during steady-state spermatogenesis and the gonocytes of neonatal testes. GFRα-1 is expressed in SSC cultured (or isolated) from neonatal, pubertal, and adult testes, as well as progenies are produced in the transplantation of gonocyte-originated SSC and SSC activity is higher in mice transplanted with GFRα-1+ cells from pubertal testes [[24](#_ENREF_24), [31-50](#_ENREF_31), [55](#_ENREF_55), [74-82](#_ENREF_74)]. Interestingly, mouse GFRα-1+ SPG used for live-image tracing experiments have shown that A_s_ SPG are fragmented from A_pr_ and A_al_ SPG in seminiferous tubules [[55](#_ENREF_55)]. Recently, enhanced repopulation of donor-derived SSC, which originated from GFRα-1+ SPG is observed in the testis of host mice via temporary suppression of SPG differentiation [[54](#_ENREF_54)]. In vertebrates, GFRα-1 is a common marker for the SSC pool, and its expression is exclusively observed in gonocytes, undifferentiated type A SPG, and cultured SSC. In addition, GFRα-1+ cells are often used for transplantation experiments to produce donor-derived offspring and for elucidating the SSC model in rodents.

**2. INTEGRIN-α6**

In chicken, integrin subunit alpha 6, (INTEGRIN-α6) is detected in SSC cultured from ESCs and PGCs, and it is used for immunocytochemistry and (FACS) [[88-94](#_ENREF_88), [96](#_ENREF_96), [97](#_ENREF_97)]. In quails, SSC, which are isolated and cultured from juvenile and adult testicular cells, express INTEGRIN-α6 [[11](#_ENREF_11)]. In mice, INTEGRIN-α6+ SPG transplanted into the testes of immune-deficient mice show high colonization efficiency with the production of offspring, and INTEGRIN-α6 has been used to characterize the SPG phenotype in SSC culture using FACS [[107-111](#_ENREF_107)]. In goats, INTEGRIN-α6 is primarily localized in the plasma membrane of SPG lying adjacent to the basement membrane of the seminiferous tubules and is expressed in cultured SSC [[20](#_ENREF_20), [67](#_ENREF_67), [102-104](#_ENREF_102)]. In bulls, INTEGRIN-α6 was detected in isolated and cultured prepubertal bovine testicular germ cells [[100](#_ENREF_100)]. In pigs, INTEGRIN-α6 expression is detected in undifferentiated SPG populations of prepubertal testes using FACS [[112](#_ENREF_112)]. In monkeys, INTEGRIN-α6 protein is observed in the basement membrane of the seminiferous tubules and is co-localized and overlapped with GFRα-1+ cells [[29](#_ENREF_29)]. In humans, INTEGRIN-α6 is abundantly expressed in SPG in the basement membrane of seminiferous tubules, and INTEGRIN-α6+ SPG isolated from adult testes can repopulate in the basement membrane of seminiferous tubules in recipient mouse testes. In addition, INTEGRIN-α6 is used for phenotypic identification in isolated and cultured SSC [[22](#_ENREF_22), [23](#_ENREF_23), [99](#_ENREF_99), [105](#_ENREF_105), [106](#_ENREF_106)]. In vertebrates, INTEGRIN-α6 is used to identify SSC phenotypes in mice and chickens more often than detecting SSCs in the testes using immunohistochemistry and immunocytochemistry.

**3. INTEGRIN-ß1**

In chicken, integrin subunit beta (INTEGRIN-ß1) is also used for SSC detection and labeling in ESCs (or PGCs), which is performed with cultured SSCs using immunocytochemistry and FACS [[89](#_ENREF_89), [91](#_ENREF_91), [92](#_ENREF_92), [94](#_ENREF_94), [96](#_ENREF_96), [97](#_ENREF_97), [113](#_ENREF_113)]. Cultured bovine SSCs are found to be INTEGRIN-ß1-positive using FACS and immunocytochemistry [[100](#_ENREF_100)]. In pigs, undifferentiated SPG from prepubertal pig testes are positive for INTEGRIN-ß1 using FACS, and this protein is detected in purified SSCs and undifferentiated SPG [[112](#_ENREF_112), [114](#_ENREF_114)]. Germ cell-derived colonies cultured from neonatal goat testes express INTEGRIN-ß1 [[101](#_ENREF_101), [103](#_ENREF_103), [104](#_ENREF_104)]. In mice, INTEGRIN-ß1+ SPG are efficiently colonized in the testes of immune-deficient mice, and SSCs cultured from prepubertal and adult testes express INTEGRIN-ß1 and are used for characterizing the SPG phenotype in in vitro SSC cultures [[82](#_ENREF_82), [107](#_ENREF_107), [108](#_ENREF_108), [111](#_ENREF_111)]. However, evidence-based on immunohistochemistry of the seminiferous tubules has not been sufficiently reported in vertebrates. INTEGRIN-ß1 is a useful marker for determining the SSC phenotype in mice and chickens.

**4. THY1**

MACS-separated SPG with thymocyte differentiation antigen 1 (THY1) antibody are cultured for an extended period in rohu and catfish and observed to differentiate into functional sperm in catfish [[115](#_ENREF_115), [116](#_ENREF_116)]. In chickens, the THY1 is localized at the basement membrane of the seminiferous tubules in mature testes [[117](#_ENREF_117)]. In bulls, THY1+ cell fractions separated from prepubertal testes with THY1 antibody, which are also positive for PLZF, generate more colonies in recipient testes following xenogeneic transplantation, and THY1 is expressed in isolated undifferentiated SPG and cultured SSCs [[13](#_ENREF_13), [100](#_ENREF_100), [119](#_ENREF_119)]. In buffaloes, THY1 is observed in undifferentiated SPG of prepubertal testes, and cultured undifferentiated SPG express THY1 [[118](#_ENREF_118)]. In goats, THY1 is detected at the basement membrane of the seminiferous tubules in prepubertal testes, and MACS-separated THY1+ cells express undifferentiated SPG markers (PLZF and PGP 9.5), cultured (or isolated) SSCs express THY1, and THY1+ cells are colonized in the seminiferous tubules of recipient mice [[101](#_ENREF_101), [103](#_ENREF_103), [120](#_ENREF_120), [124](#_ENREF_124)]. In pigs, THY1 is used for detecting undifferentiated SPG, and SSCs cultured with THY1 expression can induce spermatogenesis in vitro [[112](#_ENREF_112), [128](#_ENREF_128)]. In treeshrews, THY1 is detected at the basement membrane of the seminiferous tubules, and cultured SSCs are positive for the anti-THY1 antibody. In addition, EGFP-tagged SSCs are able to restore spermatogenesis and successfully generate transgenic offspring [[123](#_ENREF_123)]. In mice, THY1 protein is observed in the seminiferous tubules in an adult mouse testis grafted into the dorsal skin of nude mice. It is expressed to some extent in the cytoplasm of the SSC colonies, which can produce the progenies; THY1+ cells from prepubertal testes are co-stained with PLZF+ cells [[39](#_ENREF_39), [126](#_ENREF_126), [127](#_ENREF_127)]. In monkeys, THY1+ cells isolated from juvenile rhesus testes enhance colonizing activity in xenotransplantation to recipient mice, and THY1 is used for undifferentiated SPG detection in adult monkeys [[27](#_ENREF_27), [29](#_ENREF_29), [30](#_ENREF_30)]. In addition, THY1+ cells of monkeys transplanted into allogeneic recipients produce functional sperm cells that are competent for fertilization and preimplantation embryo development [[125](#_ENREF_125)]. In human adult testes, THY1 is expressed in a subpopulation of SPG, and isolated and cultured SSCs express THY1 [[22](#_ENREF_22), [73](#_ENREF_73)]. THY1 is predominantly expressed in germ cells in the lumen of the human adult seminiferous tubules, peritubular, and interstitial cells [[99](#_ENREF_99)]. Other studies on humans have reported that THY1 is expressed in fetal male (15 and 19 weeks of gestation) gonocytes during the third trimester of gestation and that THY1 expression shifts exclusively to the somatic cells of the testes, where it continues to be detected only in the somatic cells postnatally [[122](#_ENREF_122)]. A similar result has been reported wherein THY1 is expressed in human adult testicular somatic cells and demonstrates mesenchymal properties [[121](#_ENREF_121)]. In humans, THY1 expression is controversial in the seminiferous tubules of the testis. However, THY1 is often used in primates, goats, and mice for MACS and in vitro culture as a reliable undifferentiated SPG marker.

**5. SSEA-1**

In SSC cultures of rohu, the cell-surface marker stage-specific embryonic antigen-1 (SSEA-1) is expressed in THY1+ SPG, and its expression is maintained in the long-term culture of SSCs [[115](#_ENREF_115)]. In chicken, SSEA-1 is expressed in PGCs, embryonic germ cells, and cultured (or isolated) SSCs, and SSEA-1+ cells-transplanted chicken embryos and testes exhibit SSC activity by producing the progeny [[97](#_ENREF_97), [129](#_ENREF_129), [130](#_ENREF_130), [133](#_ENREF_133)]. In addition, chicken SSEA-1+ SSCs can differentiate into adipocytes, neuron-like cells, and osteoblasts in vitro, and SSEA-1 is expressed in fetal SSCs [[131](#_ENREF_131)]. SSEA-1 staining is observed in A_s_ and A_pr_ SPG located at the basement membrane of seminiferous tubules in blackbuck testes [[134](#_ENREF_134)]. In rats, SSEA-1 expression is found in migrating PGCs on different embryonic days [[137](#_ENREF_137)]. In cats, SSEA-1 is localized at the undifferentiated SPG of prepubertal and adult testes, and SSEA-1 expression is detected in undifferentiated SPG isolated from prepubertal and adult testes [[135](#_ENREF_135)]. In mice, cultured SSEA-1+ SSCs differentiate into mesoderm and neurons [[136](#_ENREF_136)]. In tammar, SSEA-1 is observed in the gonocytes of fetal gonads [[138](#_ENREF_138)]. In pigs, the enrichment of PGP 9.5+, PLZF+, and VASA+ cells is observed in SSEA-1+ cells, and xenotransplantation of SSEA-1-expressing SPG results in significantly more colonies of donor-derived germ cells [[112](#_ENREF_112)]. SSEA-1 is expressed in PGCs and undifferentiated SPG, which has been evaluated by immunohistochemistry, FACS, transplantation, and SSC culture experiments.

**6. SSEA-4**

In chicken, stage-specific embryonic antigen-4 (SSEA-4) is expressed in cultured (or isolated) SSCs from juvenile and adult testes [[133](#_ENREF_133)]. Cat SSEA-4 is localized at the basement membrane of the seminiferous tubules of adult and prepubertal testes, and its expression has been observed in SPG isolated by FACS [[135](#_ENREF_135)]. In marmosets, strong SSEA-4 expression is observed in SPG, with 8.6 ± 1.61% SSEA-4+ cells detected in adult marmoset testes using FACS [[140](#_ENREF_140)]. In lion-tailed macaques and rhesus monkeys, SSEA-4 is expressed mostly in the cytoplasm of single and paired SPG as well as in rhesus SSEA-4+ cells show higher colonization in the testes of recipient mice after transplantation [[29](#_ENREF_29), [140](#_ENREF_140)]. In humans, SSEA-4 expression is observed in SPG of the adult testes and gonocytes of fetal testes, and SSEA-4+ SSCs cultured from adult and pubertal testes maintain the expression of PLZF and OCT4. SSC-derived human teratoma formation is observed after SSC injection into an immunodeficient mouse. [[68](#_ENREF_68), [99](#_ENREF_99), [106](#_ENREF_106), [121](#_ENREF_121), [122](#_ENREF_122), [141](#_ENREF_141)]. In chickens and primates, SSEA-4 is frequently used for immunohistochemistry, FACS, transplantation, and SSC culture.

**7. PLZF**

In the immature rohu testis, promyelocytic leukemia zinc finger protein (PLZF) is mainly localized in the nuclear compartment of in vitro-propagated SSCs for two years [[143](#_ENREF_143)]. In catfish, PLZF transcript signals are found in type A undifferentiated and differentiated SPG in mature testes [[144](#_ENREF_144)]. In mature dogfish testes, PLZF expression is strong in undifferentiated SPG but weak in proliferating SPG, and cultured SPG express PLZF [[1](#_ENREF_1)]. In rainbow trout, PLZF is expressed mostly in undifferentiated type A SPG from immature testes and undifferentiated type A SPG from spawning testes [[145](#_ENREF_145)]. In adult testes of zebrafish, PLZF is localized specifically in undifferentiated type A SPG, in contrast with differentiated SPG and type B SPG which possess small particles of PLZF protein in adult testis [[146](#_ENREF_146)]. In bulls, PLZF is observed in undifferentiated SPG of prepubertal and peripubertal testes, and MACS-separated SPG (THY1+ or GFRα-1+ SPG) and SSCs cultured (or isolated) from the prepubertal testis express PLZF [[13](#_ENREF_13), [119](#_ENREF_119), [147](#_ENREF_147), [148](#_ENREF_148)]. In cats, PLZF immunostaining is observed in the gonocytes in fetal testes and SPG at the basement membrane of the seminiferous tubules in prepubertal and adult testes [[149](#_ENREF_149)]. In dogs, PLZF is detected in undifferentiated SPG of prepubertal and type A SPG of the adult testis, and SSCs cultured from prepubertal and adult testes express PLZF [[18](#_ENREF_18), [150](#_ENREF_150)]. In horses, donkeys, and mules, PLZF is detected in the nucleus of undifferentiated SPG of adult testes [[19](#_ENREF_19)]. In goats, PLZF is expressed in the SPG of adult testes and SSC colonies; these colonies are cultured and isolated from neonatal and prepubertal testes [[67](#_ENREF_67), [101](#_ENREF_101), [102](#_ENREF_102), [104](#_ENREF_104), [120](#_ENREF_120), [124](#_ENREF_124), [151](#_ENREF_151)]. In yak, all PLZF+ cells are gonocytes of the neonatal testis in the lumen and SPG of prepubertal and adult testes in the basement membrane of seminiferous tubules [[181](#_ENREF_181)]. In sheep, PLZF is expressed in undifferentiated SPG located at the basement membrane of seminiferous tubules of prepubertal testes, and PLZF is also expressed in SSC colonies cultured from prepubertal testes [[180](#_ENREF_180), [191](#_ENREF_191)]. In pigs, PLZF is expressed partially in PGP 9.5+ gonocytes of neonatal testes and PGP 9.5+ undifferentiated SPG of prepubertal and adult testes. Moreover, undifferentiated SPG, cultured (or isolated) from neonatal and prepubertal testes, express PLZF [[85](#_ENREF_85), [86](#_ENREF_86), [112](#_ENREF_112), [128](#_ENREF_128), [176](#_ENREF_176), [177](#_ENREF_177), [188](#_ENREF_188)]. In monkeys, PLZF is detected in the undifferentiated type A_dark_ and A_pale_ SPG of juvenile and adult testes [[26](#_ENREF_26), [27](#_ENREF_27)]. In humans, PLZF is observed in the nucleus of undifferentiated SPG located in the basement membrane of seminiferous tubules of adult testis. Additionally, SSCs cultured (or isolated) from the adult testes express PLZF [[22](#_ENREF_22), [30](#_ENREF_30), [69](#_ENREF_69), [73](#_ENREF_73), [105](#_ENREF_105), [152](#_ENREF_152), [182](#_ENREF_182)]. In rats, PLZF is expressed in undifferentiated SPG of prepubertal testes and undifferentiated germ cells cultured from prepubertal testes [[179](#_ENREF_179), [189](#_ENREF_189), [190](#_ENREF_190)]. In treeshrews, PLZF is detected in SSCs cultured from THY+ SPG of prepubertal and adult testes [[123](#_ENREF_123)]. In mice, the absence of PLZF causes an increase in apoptosis and subsequent loss of tubular structure. Mouse PLZF is required for SSC self-renewal and maintenance of the undifferentiated state of SSCs [[169](#_ENREF_169)]. Mouse PLZF is observed in the gonocytes of neonatal testes and undifferentiated SPG of postnatal, prepubertal, and adult testes, and it is co-expressed with OCT4+ undifferentiated SPG [[24](#_ENREF_24), [31-34](#_ENREF_31), [36](#_ENREF_36), [38](#_ENREF_38), [41](#_ENREF_41), [44](#_ENREF_44), [45](#_ENREF_45), [48](#_ENREF_48), [50](#_ENREF_50), [82](#_ENREF_82), [126](#_ENREF_126), [127](#_ENREF_127), [153-170](#_ENREF_153)]. In addition, mouse SSCs cultured (or isolated) from neonatal, pup, and adult mouse testes show PLZF expression [[34](#_ENREF_34), [41](#_ENREF_41), [74](#_ENREF_74), [75](#_ENREF_75), [79](#_ENREF_79), [80](#_ENREF_80), [82](#_ENREF_82), [126](#_ENREF_126), [154](#_ENREF_154), [156-159](#_ENREF_156), [162](#_ENREF_162), [183-186](#_ENREF_183), [223](#_ENREF_223)]. PLZF is a strong common marker for gonocytes, undifferentiated SPG, and type A SPG in fish and mammals, and it is known as an important transcription factor involved in SSC pool maintenance.

**8. NANOS genes**

In flounder, nanos C2HC-type zinc finger (NANOS) 2 protein is expressed in PGCs and undifferentiated SPG of testes from the early to the adult stage of development [[193](#_ENREF_193)]. In medaka, NANOS is expressed in the PGC of the embryo, which has been detected using NANOS-3′UTR-GFP transgenic medaka fish [[194](#_ENREF_194)]. In adult tilapia, NANOS2 expression is frequently observed in clusters of single PGCs, gonocytes, and type A undifferentiated SPG in the testis; SSCs cultured from adult testes express NANOS2 and are colonized in the testes of allogeneic recipients [[3](#_ENREF_3), [224](#_ENREF_224)]. In rainbow trout, NANOS2 transcripts are detected in gonocytes before testis differentiation and type A undifferentiated SPG from the juvenile to the spawning stage [[145](#_ENREF_145)]. In zebrafish, NANOS2 mRNA is observed in germline stem cells of the adult testis [[195](#_ENREF_195)]. In Japanese clawed frogs, NANOS3 mRNA is strongly expressed in PGCs and gonocytes of the tadpole testis [[225](#_ENREF_225)]. In goats, NANOS2 is expressed in gonocytes and SPG located near the basement membrane of seminiferous tubules of the adult testis. SSC colonies cultured from postnatal testis also express NANOS2 [[67](#_ENREF_67), [196](#_ENREF_196)]. In adult horses, the expression of NANOS2 protein is detected in the SPG of the testis and cultured SSCs [[21](#_ENREF_21)]. In particular, NANOS1 and NANOS2 expression is observed in gonocytes and undifferentiated SPG in postnatal and adult testes [[57](#_ENREF_57)]. A_s_ and A_pr_ SPG are positive for NANOS2 in cultured rat SSCs. NANOS2 is detected in undifferentiated SPG located in the basement membrane of seminiferous tubules, and THY1+ SSCs are also positive for NANOS2, which has been detected using FACS [[137](#_ENREF_137)]. In mouse adult testes, NANOS2 is expressed in A_s_ and A_pr_ SPG for maintaining the undifferentiated state of SSCs, and NANOS3 is expressed in A_pr_ and A_al_ SPG during steady-state spermatogenesis. NANOS2 and NANOS3 loss induce defective PGC and gonocyte-to-SPG development in the embryonic stage [[34](#_ENREF_34), [36](#_ENREF_36), [49](#_ENREF_49), [50](#_ENREF_50), [198](#_ENREF_198)]. In humans and marmosets, NANOS1 is expressed in gonocytes during the fetal and postnatal periods [[197](#_ENREF_197)]. In vertebrates, NANOS genes are highly expressed in PGCs, gonocytes, and undifferentiated SPG.

**9. OCT4**

In catfish, octamer-binding transcription factor 4 (OCT4) is mainly expressed in type A undifferentiated and differentiated SPG with no expression in type B SPG [[144](#_ENREF_144)]. In adult dogfish testes, the OCT4 transcript is detected in the nucleus and cytoplasm of undifferentiated single and paired SPG but not in somatic cells [[2](#_ENREF_2)]. In medaka, OCT4 is expressed in PGCS during the embryonic stage and is detected in undifferentiated SPG, which constitutes the germ stem cell population of the testis [[194](#_ENREF_194)]. In rohu, gradient- and MACS-separated THY1+ cells isolated from the prepubertal testis express OCT4 [[115](#_ENREF_115)]. Colony-forming cells cultured from the testis of newborn chickens express OCT4 and differentiate into SPZ-like cells [[199](#_ENREF_199)]. In buffaloes, OCT4 expression is observed in the gonocytes of prepubertal testes and germ cells (SPG, SPC, and STD) of the adult testis and co-stained with most DBA+ gonocytes (or SPG) in isolated cells of prepubertal testes [[200](#_ENREF_200)]. In addition, OCT4 is observed in undifferentiated SPG of prepubertal testes and cultured SPG express OCT4 [[118](#_ENREF_118), [201](#_ENREF_201)]. Cat OCT4 is expressed in PLZF+ A_s_ or A_pr_ SPG of prepubertal testes within the seminiferous tubules, and its expression pattern in the adult is limited to a subset of SPG along the basement membrane [[202](#_ENREF_202)]. In adult testes of cheetah and leopard, OCT4 expression is also limited to SPG bordering the basement membrane [[202](#_ENREF_202)]. In bulls, OCT4 is expressed in germ cell-derived colonies cultured for long- and short-term duration [[63](#_ENREF_63), [100](#_ENREF_100)]. In goats, SSCs cultured from prepubertal testes express OCT4 [[66](#_ENREF_66), [67](#_ENREF_67), [103](#_ENREF_103), [104](#_ENREF_104), [124](#_ENREF_124)]. In rats, OCT4 is detected in gonocytes and undifferentiated SPG during the fetal and neonatal periods [[197](#_ENREF_197), [206](#_ENREF_206)]. In treeshrews and lamb, OCT4 is expressed in SSCs cultured from prepubertal and adult testes [[123](#_ENREF_123), [191](#_ENREF_191)]. In rabbits, SSCs cultured (or isolated) from prepubertal testes express OCT4 [[87](#_ENREF_87)]. In marmosets, OCT4 is localized in gonocytes during the fetal and postnatal periods of the development of testes [[197](#_ENREF_197), [205](#_ENREF_205)]. In neonatal porcine testes, OCT4 is localized in the gonocytes, and OCT4+ SSCs cultured from neonatal and prepubertal testes differentiate into post-meiotic germ cells [[114](#_ENREF_114), [128](#_ENREF_128)]. In monkeys, PGCs are positive for OCT4 in embryonic testes at day 49 within the testicular cords [[204](#_ENREF_204)]. OCT4 is expressed in human gonocytes of fetal, neonatal, and postnatal testes, specifically localized at the basement membrane of the seminiferous tubules in SSEA-4+ SPG of adult testes and OCT4 is detected in SSCs cultured from fetal and adult testes [[99](#_ENREF_99), [106](#_ENREF_106), [122](#_ENREF_122), [142](#_ENREF_142), [197](#_ENREF_197), [203](#_ENREF_203)]. In mice, OCT+ cells are observed in gonocytes and SPG, and SSC colonies cultured from the adult, prepubertal, and neonatal testes show OCT4 expression [[75](#_ENREF_75), [126](#_ENREF_126), [154](#_ENREF_154), [163](#_ENREF_163), [168](#_ENREF_168), [183](#_ENREF_183), [185](#_ENREF_185), [186](#_ENREF_186)]. OCT4 is broadly expressed in gonocytes and undifferentiated SPG as a pluripotent stem cell marker in fish and mammals.

**10. DAZL**

In medaka, deleted in azoospermia-like (DAZL) transcripts are observed in PGCs during embryonic stages, and strong DAZL expression is detected in SPG located in most peripheral regions; additionally, DAZL expression reduces in SPC and disappears in the post-meiotic stage [[207](#_ENREF_207)]. In chicken, DAZL is expressed in the nucleus and cytoplasm of circulating PGCs in the embryonic, premature gonads and SPG at the basement membrane of seminiferous tubules in mature chicken testes, and SSCs cultured from embryonic PGCs also express DAZL [[91](#_ENREF_91), [97](#_ENREF_97), [130](#_ENREF_130), [208](#_ENREF_208), [209](#_ENREF_209)]. In quail, DAZL coexists with GFRα-1-expressing germ cells in isolated testicular cells, and SSCs cultured from juvenile and adult testes express DAZL [[11](#_ENREF_11)]. In dogs, DAZL-expressing SPG are located in the lumen of seminiferous tubules at the prepubertal stage and the basement membrane of seminiferous tubules at the pubertal, postpubertal, and adult stages, and DAZL protein is detected in SSCs cultured from prepubertal testes [[18](#_ENREF_18), [150](#_ENREF_150), [210](#_ENREF_210)]. In horses, DAZL is localized in the SPG and primary SPC of the postpubertal and adult testes adjacent to the basal membrane and the cytoplasm of some SPG of prepubertal and pubertal testes, and DAZL is stained in isolated SPG or primary SPC in the postpubertal stage [[211](#_ENREF_211), [212](#_ENREF_212)]. In humans, DAZL is immunolocalized to the nuclei of germ cells in the testes in the 1^st^ and 2^nd^ trimester stages, and DAZL expression from the nucleus to cytoplasm correlates with the downregulation of OCT4, and the onset of the expression of VASA as well as DAZL is detected in SSCs cultured (or isolated) from adult testes [[106](#_ENREF_106), [213](#_ENREF_213)]. In rats, DAZL is expressed in gonocytes during the embryonic stage and in long-term cultured SSCs producing their progeny in recipient rat testes [[190](#_ENREF_190), [206](#_ENREF_206)]. In mouse testes, DAZL is detected in the basement membrane of seminiferous tubules from germ cell-deficient testes injected with exogenous SSCs, and DAZL expression is observed in gonocytes, SPG, and SPC of neonatal and adult testes. Additionally, SSCs cultured from prepubertal testes express DAZL [[42](#_ENREF_42), [76](#_ENREF_76), [157](#_ENREF_157), [186](#_ENREF_186), [187](#_ENREF_187), [214](#_ENREF_214)].

**11. GPR125**

In newborn chicken, 16% of total testicular cell populations are positive for G protein-coupled receptor 125 (GPR125), and germ cell-derived colonies express GPR125 in vitro, as previously demonstrated in a chicken SSC culture [[199](#_ENREF_199)]. A large proportion of cells are positive for GFRA125 in isolates from mixed (adult and prepubertal) germ cells of cats [[135](#_ENREF_135)]. In mice, GPR125 expression is observed only in SPG of adult testes and SSC colonies cultured from knock-in mice harboring GPR125-LacZ; additionally, GPR125-expressing SPG are colonized in the testes of recipient mice and form the teratoma [[186](#_ENREF_186)]. In humans, GPR125 is expressed only in SPG within the seminiferous tubules, and cultured human SSCs from adult testes express GPR125 and differentiate into human ES-like cells [[22](#_ENREF_22), [70](#_ENREF_70), [71](#_ENREF_71), [73](#_ENREF_73), [141](#_ENREF_141), [182](#_ENREF_182)]. GPR125 expression is limited to SPG in birds and mammals and is often used for performing FACS in humans.

**12. DND**

In adult testes of turbot, transcripts of dead-end protein (DND) homologous genes are predominantly detected in SPG, and their levels are decreased in SPC [[217](#_ENREF_217)]. In medaka, DND mRNA is expressed in the SPG of the testicular periphery, while its expression is reduced in SPC and STD. Moreover, DND expression is observed in embryonic PGCs [[216](#_ENREF_216)]. However, tuna DND mRNA expression is restricted to PGCs, gonocytes, and type A SPG in larvae, juvenile, immature, and mature testes, and it is not detected in other differentiated spermatogenic cells [[215](#_ENREF_215)]. In mice, DND1 is expressed in a subpopulation of differentiating and undifferentiated SPG, and it has a significant role in the differentiation of SPG. Chicken DND homologs are detected in chicken VASA+ cells of premature and adult testes [[208](#_ENREF_208), [218](#_ENREF_218)]. Avian and mammalian DND expression is observed mainly in SPG and differentiated meiotic cells, but it is expressed in undifferentiated germ cells of fish. Further studies on DND are necessary to identify its expression pattern in testicular germ cells.

**13. NANOG**

In medaka, the Nanog homeobox (NANOG) mRNA signal is observed only in the SPG of adult testes using in situ hybridization [[219](#_ENREF_219)]. In blackbuck, NANOG is expressed in undifferentiated SPG and SPC, and a few SPG are negative for NANOG immunostaining [[134](#_ENREF_134)]. In adult human testes, NANOG expression is co-localized with SSEA-4+ cells repopulated in the testes of busulfan-treated nude mice [[99](#_ENREF_99)]. In mice, rare NANOG+ cells are detected in SPG and SPC of adult testes, and NANOG-overexpressing SPG (collected from 6-day old mice) promote the proliferation of cultured SSCs in vitro and regenerate normal colonies of SSC in the testes of recipient mice [[220](#_ENREF_220)]. Bovine NANOG is expressed in colonies derived from the long-term culture of male gonocytes [[63](#_ENREF_63)]. In goats, NANOG is detected in putative SSC colonies derived from gonocytes on the 12th culture day [[67](#_ENREF_67), [124](#_ENREF_124)]. It is necessary to identify the localization of NANOG in other species.

**14. CSF1R AND TRA-1-81**

In scorpion mud turtle testes, all undifferentiated type A SPG are positive for colony-stimulating factor 1 receptor (CSF1R) and are predominantly located in regions where seminiferous tubules face the interstitial compartment containing blood vessels [[8](#_ENREF_8)]. In donkeys, horses, and mules, CSF1R is expressed in undifferentiated type A SPG (A_s_ and A_pr_) of seminiferous tubules [[19](#_ENREF_19)]. In rohu, TRA-1-81 is expressed in MACS-separated SPG using mouse anti-THY1.2 MicroBeads [[115](#_ENREF_115)]. In cats, podocalyxin-like protein-1 (TRA-1-81 or PCLP-1) is found in pairs and chains of SPG localized at the basement membrane of the seminiferous tubules, and it is expressed in isolated SPG [[135](#_ENREF_135)]. TRA-1-81 is expressed in type A undifferentiated SPG of adult marmosets, lion-tailed macaques, and rhesus monkeys [[140](#_ENREF_140)]. CSF1R and TRA-1-81 are expressed in undifferentiated SPG only in a few species.

**Supplementary Table 2.** Molecular markers on the spermatogonial stem cell pool and type A spermatogonia in testes and cultured (or isolated) spermatogonia in mammals.

| **Subgroups**  **Markers** | **Mammals** | |
| --- | --- | --- |
|  | **Testis tissue** | **In vitro** |
| **PGP 9.5 (UCHL1)** | Buffalo [[118](#_ENREF_118), [201](#_ENREF_201), [226](#_ENREF_226)]  Bull [[13](#_ENREF_13), [119](#_ENREF_119), [227-230](#_ENREF_227)]  Cat [[202](#_ENREF_202), [231](#_ENREF_231), [232](#_ENREF_232)]  Cheetah [[202](#_ENREF_202)]  Dog [[150](#_ENREF_150), [210](#_ENREF_210), [233-236](#_ENREF_233)]  Goat [[191](#_ENREF_191)]  Human [[22](#_ENREF_22), [25](#_ENREF_25), [237](#_ENREF_237)]  Leopard [[202](#_ENREF_202)]  Monkey [[30](#_ENREF_30)]  Mouse [[238](#_ENREF_238)]  Pig [[58](#_ENREF_58), [59](#_ENREF_59), [83](#_ENREF_83), [86](#_ENREF_86), [112](#_ENREF_112), [128](#_ENREF_128), [176-178](#_ENREF_176), [188](#_ENREF_188), [239-249](#_ENREF_239)]  Sheep [[250](#_ENREF_250), [251](#_ENREF_251)] | Buffalo [[118](#_ENREF_118), [201](#_ENREF_201)]  Bull [[13](#_ENREF_13), [63-65](#_ENREF_63), [228](#_ENREF_228), [230](#_ENREF_230)]  Dog [[150](#_ENREF_150), [233](#_ENREF_233), [236](#_ENREF_236)]  Goat [[124](#_ENREF_124), [252](#_ENREF_252), [253](#_ENREF_253)]  Human [[69](#_ENREF_69), [73](#_ENREF_73), [141](#_ENREF_141), [237](#_ENREF_237), [254](#_ENREF_254)]  Pig [[58](#_ENREF_58), [59](#_ENREF_59), [84-86](#_ENREF_84), [112](#_ENREF_112), [114](#_ENREF_114), [128](#_ENREF_128), [176](#_ENREF_176), [177](#_ENREF_177), [240](#_ENREF_240), [241](#_ENREF_241), [245](#_ENREF_245), [247-249](#_ENREF_247), [255](#_ENREF_255)] |
| **SALL4** | Baboon [[256](#_ENREF_256)]  Cat [[256](#_ENREF_256)]  Dog [[256](#_ENREF_256)]  Human [[256](#_ENREF_256)]  Mandrill [[256](#_ENREF_256)]  Marmose t[[205](#_ENREF_205), [256](#_ENREF_256)]  Monkey [[256](#_ENREF_256)]  Mouse [[42](#_ENREF_42), [44](#_ENREF_44), [45](#_ENREF_45), [53](#_ENREF_53), [81](#_ENREF_81), [164](#_ENREF_164), [256](#_ENREF_256), [257](#_ENREF_257)]  Pig [[258](#_ENREF_258)]  Treeshrew [[256](#_ENREF_256)] | Monkey [[125](#_ENREF_125)]  Mouse [[81](#_ENREF_81), [257](#_ENREF_257)] |
| **DBA** | Blackbuck[[134](#_ENREF_134)]  Buffalo[[118](#_ENREF_118)]  Bull[[147](#_ENREF_147), [228](#_ENREF_228), [259](#_ENREF_259), [260](#_ENREF_260)]  Cat[[232](#_ENREF_232)]  Pig[[178](#_ENREF_178), [248](#_ENREF_248), [261](#_ENREF_261)]  Rat[[206](#_ENREF_206)] | Alpaca[[262](#_ENREF_262), [263](#_ENREF_263)]  Buffalo[[200](#_ENREF_200), [201](#_ENREF_201)]  Bull[[63](#_ENREF_63), [65](#_ENREF_65), [100](#_ENREF_100), [147](#_ENREF_147), [228](#_ENREF_228), [230](#_ENREF_230), [264](#_ENREF_264)]  Goat[[101](#_ENREF_101)]  Pig[[128](#_ENREF_128), [248](#_ENREF_248), [255](#_ENREF_255), [265](#_ENREF_265)] |
| **CXCR4** | Bull [[266](#_ENREF_266)]  Dog [[234](#_ENREF_234)]  Goa t[[151](#_ENREF_151)]  Mouse [[155](#_ENREF_155)]  Pig [[241](#_ENREF_241)]  Yak [[181](#_ENREF_181)] | Mouse [[155](#_ENREF_155)]  Pig [[241](#_ENREF_241)] |
| **LIN28** | Dog [[234](#_ENREF_234)]  Horse [[267](#_ENREF_267)]  Marmoset [[205](#_ENREF_205)]  Mouse [[38](#_ENREF_38), [42](#_ENREF_42), [48](#_ENREF_48), [80](#_ENREF_80), [157](#_ENREF_157), [185](#_ENREF_185), [268](#_ENREF_268)]  Human [[237](#_ENREF_237)] | Goat [[67](#_ENREF_67)]  Mouse [[185](#_ENREF_185)] |
| **UTF1** | Horse [[269](#_ENREF_269)]  Human [[25](#_ENREF_25)]  Monkey [[270](#_ENREF_270)]  Pig [[242](#_ENREF_242)] | Human [[182](#_ENREF_182), [254](#_ENREF_254)]  Pig [[242](#_ENREF_242)] |
| **CD9** | Human [[23](#_ENREF_23)]  Mouse [[82](#_ENREF_82), [271](#_ENREF_271)] | Human [[68](#_ENREF_68)]  Mouse [[39](#_ENREF_39), [108](#_ENREF_108), [111](#_ENREF_111), [271](#_ENREF_271)]  Rat [[271](#_ENREF_271)] |
| **MAGEA4** | Monkey [[270](#_ENREF_270)]  Human [[23](#_ENREF_23), [25](#_ENREF_25), [272-274](#_ENREF_272)]  Marmoset [[197](#_ENREF_197), [205](#_ENREF_205), [275](#_ENREF_275)] | Human [[22](#_ENREF_22)]  Monkey [[270](#_ENREF_270)]  Marmoset [[205](#_ENREF_205)] |
| **ID4** | Human [[182](#_ENREF_182)]  Mouse [[78](#_ENREF_78), [170](#_ENREF_170), [276](#_ENREF_276), [277](#_ENREF_277)] | Mouse [[276](#_ENREF_276)] |
| **PAX7** | Baboon mouse [[278](#_ENREF_278)]  Bull [[278](#_ENREF_278)]  Cat [[278](#_ENREF_278)]  Deer mouse [[278](#_ENREF_278)]  Dog [[278](#_ENREF_278)]  Mouse [[278](#_ENREF_278)]  Pig [[279](#_ENREF_279)] | Pig [[279](#_ENREF_279)] |
| **SOX2** |  | Buffalo [[280](#_ENREF_280)]  Bull [[65](#_ENREF_65)]  Goat [[124](#_ENREF_124)] |

*Expression of markers in testis section and cultured (or isolated) spermatogonia is separated in this table.

**1. PGP 9.5**

Ubiquitin C-terminal hydrolase L1 (UCHL1, also known as PGP 9.5 ) is expressed in undifferentiated SPG in the prepubertal and adult testes of buffalo [[226](#_ENREF_226)]. In prepubertal and adult cat testes, PGP 9.5 protein is observed only in undifferentiated SPG within the seminiferous tubules and co-expressed with PLZF or OCT4 [[202](#_ENREF_202)]. In contrast with other reports, cat PGP 9.5 is expressed in undifferentiated SPG in adult testes, and its expression is lower in SPC, as well as also observed in the gonocytes of immature testes; additionally, undifferentiated SPG in cultured testis fragments are positive for PGP 9.5 [[202](#_ENREF_202), [231](#_ENREF_231), [232](#_ENREF_232)]. In cheetah and leopard adult testes, PGP 9.5 is expressed in undifferentiated SPG in the basement membrane of seminiferous tubules [[202](#_ENREF_202)]. In sheep, PGP 9.5 expression is observed in gonocytes and undifferentiated SPG in prepubertal and peripubertal testes. It is also detected in the SPG of the developing testis from testicular cells xenografted into the dorsal skin of immunodeficient mice [[250](#_ENREF_250), [251](#_ENREF_251)]. In dogs, PGP 9.5 expression is observed in gonocytes and undifferentiated SPG of neonatal, prepubertal, and adult testes in the seminiferous tubules. In addition, germ cell-derived colonies cultured from gonocytes of neonatal pig testis are positive for PGP 9.5 and they are colonized in the seminiferous tubules of sterile nude mice as well as a mixture of germ cell-derived colonies and testicular somatic cells, xenografted into the dorsal skin of sterile nude mice, can reconstruct the seminiferous tubules [[150](#_ENREF_150), [210](#_ENREF_210), [233-236](#_ENREF_233)]. In goats, SSCs cultured (or isolated) from prepubertal testes express PGP 9.5 [[124](#_ENREF_124), [252](#_ENREF_252), [253](#_ENREF_253)]. In buffalo, PGP 9.5 expression is detected in undifferentiated SPG of prepubertal and adult testes as well as in colonies cultured from prepubertal testes [[118](#_ENREF_118), [201](#_ENREF_201), [226](#_ENREF_226)]. In testis sections, bull PGP 9.5 is detected in gonocytes, undifferentiated SPG, THY1+ SPG, and postnatal and prepubertal testes [[13](#_ENREF_13), [119](#_ENREF_119), [227-230](#_ENREF_227)]. Undifferentiated SPG and gonocytes isolated from prepubertal bull testes are colonized in germ cell-deficient recipients and enriched undifferentiated SPG and SPG-derived colonies express PGP 9.5 in long-term culture [[13](#_ENREF_13), [63-65](#_ENREF_63), [228](#_ENREF_228), [230](#_ENREF_230)]. In monkeys, PGP 9.5 is expressed in subpopulations of spermatogonia and cultured spermatogonia [[30](#_ENREF_30)]. In humans, PGP 9.5 is localized in the cytoplasm of human SPG (including A_dark_ and A_pale_) along the basement membrane of seminiferous tubules but not in Sertoli cells or differentiated germ cells, and PGP 9.5 is expressed in cultured (or isolated) SSCs as well as it is expressed in gonocyte and A_dark_ spermatogonia of infant boys[[22](#_ENREF_22), [69](#_ENREF_69), [73](#_ENREF_73), [141](#_ENREF_141), [237](#_ENREF_237), [254](#_ENREF_254)]. In pigs, PGP 9.5 is detected in gonocytes of neonatal testes, undifferentiated SPG of prepubertal and adult testes, and cultured (or isolated) SSCs. SSCs colonized in the seminiferous tubules of sterile nude mice are positive for PGP 9.5 [[58](#_ENREF_58), [83](#_ENREF_83), [85](#_ENREF_85), [86](#_ENREF_86), [112](#_ENREF_112), [114](#_ENREF_114), [128](#_ENREF_128), [177](#_ENREF_177), [178](#_ENREF_178), [188](#_ENREF_188), [239-249](#_ENREF_239), [255](#_ENREF_255), [281](#_ENREF_281)]. In addition, porcine cultured SSCs can differentiate into haploid testicular germ cells in vitro [[83](#_ENREF_83), [128](#_ENREF_128)]. In prepubertal mouse testes, PGP 9.5 expression is only detected in the SPG of germ cells, and it appears not only in SPG but also in Sertoli cells and the adult testis [[238](#_ENREF_238)]. However, studies on mouse PGP 9.5 are limited, despite the mouse being the most studied animal for SSCs. To date, it is apparent that PGP 9.5 expression is mostly observed in gonocytes and undifferentiated SPG in mammals.

**2. SALL4**

In marmosets and mice, spalt like transcription factor 4 (SALL4) is expressed in late PGCs, gonocytes, pre-SPG, and type A SPG in fetal, newborn, prepubertal, pubertal, and adult marmoset testes, and SALL4 is expressed in cultured SSCs in mice [[42](#_ENREF_42), [44](#_ENREF_44), [45](#_ENREF_45), [81](#_ENREF_81), [164](#_ENREF_164), [205](#_ENREF_205), [256](#_ENREF_256), [257](#_ENREF_257)]. In monkeys, SALL4+ cells are found in THY1+ cells, which develop into functional sperms after allergenic transplantation [[125](#_ENREF_125)]. In humans, SALL4 protein signals are detected in gonocytes, pre-SPG, and type A SPG in fetal, prepubertal, and adult testes, respectively [[256](#_ENREF_256)]. In addition, strong SALL4 expression is conserved in undifferentiated SPG of adult Old World monkey, treeshrew, mandrill, cat, and dog testes [[256](#_ENREF_256)]. In pigs, SALL4 is expressed in undifferentiated SPG and differentiated SPG in prepubertal and pubertal testes. SSCs cultured from GFRα-1+ gonocytes express SALL4 in PGP 9.5+ colonies [[258](#_ENREF_258)]. SALL4 expression is commonly observed in undifferentiated SPG of primates and mice.

**3. DBA**

Dolichos biflorus agglutinin (DBA) protein is detected in bovine gonocytes and type A SPG until the first 30 weeks after birth [[118](#_ENREF_118), [147](#_ENREF_147), [228](#_ENREF_228), [259](#_ENREF_259), [260](#_ENREF_260)]. In bovine SSC culture, germ cell-derived colonies (or isolated SPG) express DBA in type A SPG, and DBA+ gonocytes are colonized in the seminiferous tubules of germ cell-deficient mice [[63](#_ENREF_63), [65](#_ENREF_65), [100](#_ENREF_100), [200](#_ENREF_200), [201](#_ENREF_201), [228](#_ENREF_228), [230](#_ENREF_230), [264](#_ENREF_264)]. Cat DBA is detected in undifferentiated SPG (A_s_, A_pr_, and A_al_) [[232](#_ENREF_232)]. In pigs, DBA expression is detected in gonocytes and SPG in neonatal and pubertal testes, respectively, and it is observed in isolated gonocytes and cultured putative SSCs [[128](#_ENREF_128), [178](#_ENREF_178), [248](#_ENREF_248), [255](#_ENREF_255), [261](#_ENREF_261), [265](#_ENREF_265)]. DBA protein is specifically observed in undifferentiated SPG of adult blackbuck testes [[134](#_ENREF_134)]. DBA protein is observed in gonocytes of male rat neonates [[206](#_ENREF_206)]. In neonatal male goats, DBA is expressed in germ cell-derived colonies in vitro, and cultured SSCs which are transplanted into germ cell-deficient mice are positive for DBA [[101](#_ENREF_101)]. In adult alpaca, SSCs cultured after FACS are DBA-positive [[262](#_ENREF_262), [263](#_ENREF_263)]. Generally, DBA expression is useful for undifferentiated SPG and gonocytes among domestic animals.

**4. CXCR4**

In prepubertal calves, C-X-C motif chemokine receptor 4 (CXCR4) protein is detected only in certain cells of the seminiferous tubules with higher expression of THY1 and OCT4, and MACS-separated CXCR4+ cells colonize in the testes of germ cell-deficient mice [[266](#_ENREF_266)]. In dogs, CXCR4 is expressed in the gonocytes of neonates and undifferentiated SPG near the basement membrane of seminiferous tubules in mature testes [[234](#_ENREF_234)]. CXCR4 expression correlates with that of PLZF in the membranes of SPG in dairy goat testes [[151](#_ENREF_151)]. In mice, CXCR4 expression is observed on the surface of all PLZF-expressing SPG in pup testes, but only half of PLZF-expressing SPG co-stain for CXCR4 and CXCR4 staining is also observed in ID4-expressing SPG [[223](#_ENREF_223)]. In addition, CXCR4 expression is high in THY1+ SPG and CXCR4+ cells colonize in the seminiferous tubules of germ cell-deficient nude mice [[223](#_ENREF_223)]. CXCR4 is expressed in gonocytes and undifferentiated SPG in yak testes [[181](#_ENREF_181)]. Porcine CXCR4 expression is demonstrated only in gonocytes in the seminiferous cord of neonatal testes and SSCs cultured from neonatal testes that express CXCR4 [[241](#_ENREF_241)]. In mammals, the expression of CXCR4 is seen in gonocytes and undifferentiated SPG.

**5. LIN28**

In pigs, lin-28 homolog (LIN28) expression is observed in gonocytes, undifferentiated SPG, and meiotic germ cells in neonatal, prepubertal, and postpubertal testes, respectively [[234](#_ENREF_234)]. In horses, LIN28 is expressed in A_s_ and A_pr_ SPG of pre-and postpubertal testes, and a single spermatogonium isolated from pre-and postpubertal testes expresses LIN28 [[267](#_ENREF_267)]. In goats, SSCs cultured from prepubertal testes express LIN28 [[67](#_ENREF_67)]. In neonatal and adult marmosets, LIN28 is detected in gonocytes and undifferentiated SPG [[67](#_ENREF_67)]. In mice, LIN28 is observed in undifferentiated SPG (A_s_, A_pr_, and A_al_) of adult testes and gonocytes of neonatal and prepubertal testes; in addition, cultured SSCs express LIN28 [[38](#_ENREF_38), [42](#_ENREF_42), [48](#_ENREF_48), [80](#_ENREF_80), [157](#_ENREF_157), [185](#_ENREF_185), [268](#_ENREF_268)]. In infants boys, around 60% of A_dark_ spermatogonia express LIN28 in the testis and cultured SSCs [[237](#_ENREF_237)].

**6. UTF1**

The undifferentiated embryonic cell transcription factor 1 (UFT1) gene is found only in placental mammals. In prepubertal, postpubertal, and adult pigs and stallions, UTF1 is expressed in gonocytes and SPG, and SSCs cultured from porcine neonates express UTF1 [[242](#_ENREF_242), [269](#_ENREF_269)]. In monkeys, UTF1 expression is observed in undifferentiated SPG located in the basement membrane of seminiferous tubules of adult testes [[270](#_ENREF_270)]. In adult humans, UTF1 is localized to the nuclei of SPG adjacent to the basement membrane of the seminiferous tubules and is used as a marker for SPG in the culture for human SSC enrichment [[25](#_ENREF_25), [182](#_ENREF_182), [254](#_ENREF_254)].

**7. CD9**

In humans, CD9 molecule (CD9) is expressed in undifferentiated SPG co-stained with other putative SSC markers (GFRα-1, INTEGRIN-α6, and MAGEA4), and CD9+ cells transplanted into germ cell-deficient mice are repopulated in the testis and used as a marker in SSC culture [[23](#_ENREF_23), [68](#_ENREF_68)]. In rodents, higher SSC activity is tested in transplantation with isolated CD9+ cells, and CD9 is found in the basement membrane of mouse seminiferous tubules and cultured germinal stem cells [[82](#_ENREF_82), [108](#_ENREF_108), [271](#_ENREF_271)].

**8. MAGEA4**

The melanoma-associated antigen 4 (MAGEA4) is found only in mammals, and its studies have been particularly performed in primates. In the testes of adult macaques, MAGEA4 is expressed in SPG located in the basement membrane of seminiferous tubules, SPC with weak expression, and SPG cultured with testicular cells [[270](#_ENREF_270)]. In adult marmosets, MAGEA4 is expressed in the basement membrane of adult testicular seminiferous tubules and cultured SPG, which can be repopulated in recipient mice testis, and germ cell development is detected with anti-MAGEA4 antibody in fragments of prepubertal testes xenografted into the dorsal skin of immunodeficient mice. MAGEA4 is expressed in gonocytes during the fetal and postnatal periods [[197](#_ENREF_197), [205](#_ENREF_205), [275](#_ENREF_275)]. In humans, immunohistochemical staining for MAGEA4 expression is observed in gonocytes, SPG, and SPC human testicular tissues as well as co-stained with GFRα-1+, CD9+, and INTEGRIN-α6+ cells [[22](#_ENREF_22), [23](#_ENREF_23), [25](#_ENREF_25), [272](#_ENREF_272), [274](#_ENREF_274)]. In transplantation experiments, MAGEA4 is used for detecting human germ cells in recipient mouse testes [[23](#_ENREF_23), [272](#_ENREF_272), [273](#_ENREF_273)]. MAGEA4 is often used for detecting SPG during xenotransplantation in primates.

**9. ID4**

Recently, ID4 has been highlighted as a rodent SSC marker. In mice, it has been reported that ID4+ cells exist primarily as a subset of the type A single SPG pool with higher expression, and ID4 is expressed in gonocytes and undifferentiated populations of SPG from neonatal to the adult period [[78](#_ENREF_78), [170](#_ENREF_170), [276](#_ENREF_276), [277](#_ENREF_277)]. In humans, ID4 expression, which is localized in the cells lining the basement membrane in conjunction with nuclear morphology, is observed in type A SPG of normal adult testes and overlaps with PLZF expression [[182](#_ENREF_182)]. Mouse ID4 appears to be one of the most promising putative SSC markers. However, additional studies on ID4 should be performed in other species and classes to understand its role as an SSC-specific marker.

**10. PAX7 AND SOX2**

Mouse paired box 7 (PAX7) is expressed in a rare subpopulation of A_s_ SPG of the adult testis and the percentage of PAX7+ cells in neonatal testes is higher than that in adult testes, and in a previous lineage-tracing experiment, PAX7+ cells help recover spermatogenesis in germ cell-deficient testes and produce expanding clones that gave rise to mature SPZ [[278](#_ENREF_278)]. In addition, PAX7 expression is observed and conserved in the single SPG of adult testes of cats, dogs, deer, mice, bulls, baboons, and humans as well as in the single SPG of juvenile cats and baboon testes [[278](#_ENREF_278)]. Porcine PAX7 is expressed in gonocytes and undifferentiated SPG of neonatal and prepubertal testes, co-stained with PGP 9.5+ or GFRα-1+ SPG, but not in the subsequent puberty stage, and its expression is also observed in SSCs cultured from neonatal testicular cells [[279](#_ENREF_279)]. Similar to ID4, PAX7 has been recently highlighted as a new marker of A_s_ SPG, but it still needs more studies on other species. SRY-box transcription factor 2 (SOX2) as a pluripotency marker is detected in cultured SSCs of prepubertal buffalo, goat, and cattle testes [[65](#_ENREF_65), [124](#_ENREF_124), [280](#_ENREF_280)].

**References**

1. Gautier A, Bosseboeuf A, Auvray P, Sourdaine P: **Maintenance of potential spermatogonial stem cells in vitro by GDNF treatment in a chondrichthyan model (Scyliorhinus canicula L.)**. Biology of Reproduction 2014, **91**(4).

2. Bosseboeuf A, Gautier A, Auvray P, Mazan S, Sourdaine P: **Characterization of spermatogonial markers in the mature testis of the dogfish (Scyliorhinus canicula L.)**. Reproduction 2014, **147**(1):125.

3. Santos Nassif Lacerda SM, Costa GMJ, da Silva MdA, Almeida Campos-Junior PH, Segatelli TM, Peixoto MTD, Resende RR, de França LR: **Phenotypic characterization and in vitro propagation and transplantation of the Nile tilapia (Oreochromis niloticus) spermatogonial stem cells**. General and Comparative Endocrinology 2013, **192**:95-106.

4. Nakajima S, Hayashi M, Kouguchi T, Yamaguchi K, Miwa M, Yoshizaki G: **Expression patterns of gdnf and gfrα1 in rainbow trout testis**. Gene Expression Patterns 2014, **14**(2):111-120.

5. Yang Zhao ZY, Yuan Wang, Yubing Luo, Fan Da, Wenjing Tao,Linyan Zhou, Deshou Wang, and Jing Wei: **Both Gfrα1a and Gfrα1b Are Involved in the Self-Renewal and Maintenance of Spermatogonial Stem Cells in Medaka**. Stem Cells and Development 2018, **27**(23):1658-1670.

6. Bellaïche J, Goupil A-S, Sambroni E, Lareyre J-J, Le Gac F: **Gdnf-Gfra1 pathway is expressed in a spermatogenetic-dependent manner and is regulated by fsh in a fish testis**. Biology of Reproduction 2014, **91**(4).

7. Caneguim BH, Beltrame FL, da Luz JS, Valentini SR, Cerri PS, Sasso-Cerri E: **Primordial germ cells (spermatogonial stem cells) of bullfrogs express sex hormone-binding globulin and steroid receptors during seasonal spermatogenesis**. Cells Tissues Organs 2013, **197**(2):136-144.

8. Costa GMJ, Sousa AL, Figueiredo AFA, Lacerda SMSN, França LR: **Characterization of spermatogonial cells and niche in the scorpion mud turtle (Kinosternon scorpioides)**. General and Comparative Endocrinology 2019, **273**:163-171.

9. Mucksová J, Kalina J, Bakst M, Yan H, J.P.Brillard, Benešová B, Fafílek B, Hejnar J, Trefil P: **Expression of the chicken GDNF family receptor α-1 as a marker of spermatogonial stem cells**. Animal Reproduction Science 2013, **142**(1):75-83.

10. Cheng P-L, Wu H-R, Li C-Y, Chen C-F, Cheng H-C: **Characterization of the testicular regeneration potential in premature cockerels**. Journal of Reproduction and Development 2017, **63**(6):563-570.

11. Pramod RK, Lee BR, Kim YM, Lee HJ, Park YH, Ono T, Lim JM, Han JY: **Isolation, characterization, and in vitro culturing of spermatogonial stem cells in Japanese quail (Coturnix japonica)**. Stem Cells and Development 2017, **26**(1):60-70.

12. Kim JH, Sharma N, Kim SW, Sodhi S, Ghosh M, Kim N, Mongre R, Oh S, Jeong D: **Establishment of a pheasant (Phasianus colchicus) spermatogonial stem cell line for the production of interspecies germ line chimeras**. Electronic Journal of Biotechnology 2014, **17**.

13. Kim Y-H, Choi Y-R, Kim B-J, Jung S-E, Kim S-M, Jin J-H, Yun M-H, Kim S-U, Kim Y-H, Hwang S et al: **GDNF family receptor alpha 1 is a reliable marker of undifferentiated germ cells in bulls**. Theriogenology 2019, **132**:172-181.

14. Cai H, Tang B, Wu JY, Zhao XX, Wang ZZ, An XL, Lai LX, Li ZY, Zhang XM: **Enrichment and in vitro features of the putative gonocytes from cryopreserved testicular tissue of neonatal bulls**. Andrology 2016, **4**(6):1150-1158.

15. Jiang Y, Zhu W-Q, Zhu X-C, Cai N-N, Yang R, Cai H, Zhang X-M: **Cryopreservation of calf testicular tissues with knockout serum replacement**. Cryobiology 2020, **92**:255-257.

16. Tiptanavattana N, Thongkittidilok C, Techakumphu M, Tharasanit T: **Characterization and In Vitro Culture of Putative Spermatogonial Stem Cells Derived from Feline Testicular Tissue**. Journal of Reproduction and Development 2013, **59**(2):189-195.

17. Tiptanavattana N, Techakumphu M, Tharasanit T: **Simplified isolation and enrichment of spermatogonial stem-like cells from pubertal domestic cats (Felis catus)**. Journal of Veterinary Medical Science 2015, **77**(11):1347-1353.

18. Pieri N, Souza A, Mançanares A, Roballo K, Casals J, Ambrosio C, Martins D: **Immunolocalization of proteins in the spermatogenesis process of canine**. Reproduction in Domestic Animals 2017, **52**(S2):170-176.

19. Costa GMJ, Avelar GF, Rezende-Neto JV, Campos-Junior PHA, Lacerda SMSN, Andrade BSC, Thomé RG, Hofmann M-C, Franca LR: **Spermatogonial stem cell markers and niche in equids**. PLoS One 2012, **7**(8):e44091.

20. Wu J, Liao M, Zhu H, Kang K, Mu H, Song W, Niu Z, He X, Bai C, Li G et al: **CD49f-positive testicular cells in Saanen dairy goat were identified as spermatogonia-like cells by miRNA profiling analysis**. Journal of Cellular Biochemistry 2014, **115**(10):1712-1723.

21. Costa GMJ, Avelar GF, Lacerda SMSN, Figueiredo AFA, Tavares AO, Rezende-Neto JV, Martins FGP, França LR: **Horse spermatogonial stem cell cryopreservation: feasible protocols and potential biotechnological applications**. Cell and Tissue Research 2017, **370**(3):489-500.

22. He Z, Kokkinaki M, Jiang J, Dobrinski I, Dym M: **Isolation, Characterization, and Culture of Human Spermatogonia**. Biology of Reproduction 2010, **82**(2):363-372.

23. Zohni K, Zhang X, Tan SL, Chan P, Nagano M: **CD9 is expressed on human male germ cells that have a long-term repopulation potential after transplantation into mouse testes**. Biology of Reproduction 2012, **87**(2):27.

24. Grisanti L, Falciatori I, Grasso M, Dovere L, Fera S, Muciaccia B, Fuso A, Berno V, Boitani C, Stefanini M et al: **Identification of spermatogonial stem cell subsets by morphological analysis and prospective isolation**. Stem Cells 2009, **27**(12):3043-3052.

25. Di Persio S, Saracino R, Fera S, Muciaccia B, Esposito V, Boitani C, Berloco BP, Nudo F, Spadetta G, Stefanini M et al: **Spermatogonial kinetics in humans**. Development 2017, **144**(19):3430-3439.

26. Hermann BP, Sukhwani M, Lin C-C, Sheng Y, Tomko J, Rodriguez M, Shuttleworth JJ, McFarland D, Hobbs RM, Pandolfi PP et al: **Characterization, cryopreservation, and ablation of spermatogonial stem cells in adult rhesus macaques**. Stem Cells 2007, **25**(9):2330-2338.

27. Hermann BP, Sukhwani M, Simorangkir DR, Chu T, Plant TM, Orwig KE: **Molecular dissection of the male germ cell lineage identifies putative spermatogonial stem cells in rhesus macaques**. Human Reproduction 2009, **24**(7):1704-1716.

28. Fayomi AP, Peters K, Sukhwani M, Valli-Pulaski H, Shetty G, Meistrich ML, Houser L, Robertson N, Roberts V, Ramsey C et al: **Autologous grafting of cryopreserved prepubertal rhesus testis produces sperm and offspring**. Science 2019, **363**(6433):1314-1319.

29. Maki CB, Pacchiarotti J, Ramos T, Pascual M, Pham J, Kinjo J, Anorve S, Izadyar F: **Phenotypic and molecular characterization of spermatogonial stem cells in adult primate testes**. Human reproduction 2009, **24**(6):1480-1491.

30. Mao G-P, Niu M-H, Cui Y-H, Tang R-L, Chen W, Liu B, He Z: **Characterization, isolation, and culture of spermatogonial stem cells in Macaca fascicularis**. Asian Journal of Andrology 2021, **23**(3):240-248.

31. Ishii K, Kanatsu-Shinohara M, Shinohara T: **Cell-cycle-dependent colonization of mouse spermatogonial stem cells after transplantation into seminiferous tubules**. The Journal of reproduction and development 2014, **60**(1):37-46.

32. Sharma M, Braun RE: **Cyclical expression of GDNF is required for spermatogonial stem cell homeostasis**. Development 2018, **145**(5).

33. Hasegawa K, Saga Y: **FGF8-FGFR1 signaling acts as a niche factor for maintaining undifferentiated spermatogonia in the mouse**. Biology of Reproduction 2014, **91**(6):145.

34. Niimi Y, Imai A, Nishimura H, Yui K, Kikuchi A, Koike H, Saga Y, Suzuki A: **Essential role of mouse Dead end1 in the maintenance of spermatogonia**. Developmental Biology 2019, **445**(1):103-112.

35. Grasso M, Fuso A, Dovere L, de Rooij DG, Stefanini M, Boitani C, Vicini E: **Distribution of GFRA1-expressing spermatogonia in adult mouse testis**. Reproduction 2012, **143**(3):325-332.

36. Pui HP, Saga Y: **NANOS2 acts as an intrinsic regulator of gonocytes-to-spermatogonia transition in the murine testes**. Mechanisms of development 2018, **149**:27-40.

37. Uchida A, Kishi K, Aiyama Y, Miura K, Takase HM, Suzuki H, Kanai-Azuma M, Iwamori T, Kurohmaru M, Tsunekawa N et al: **In vivo dynamics of GFRalpha1-positive spermatogonia stimulated by GDNF signals using a bead transplantation assay**. Biochemical and Biophysical Research Communications 2016, **476**(4):546-552.

38. Sharma M, Srivastava A, Fairfield HE, Bergstrom D, Flynn WF, Braun RE: **Identification of EOMES-expressing spermatogonial stem cells and their regulation by PLZF**. eLife 2019, **8**.

39. Lim JJ, Seol DW, Choi KH, Shin DH, Kim HJ, Song S-H, Lee DR: **Spermatogonial stem cell enrichment using simple grafting of testis and in vitro cultivation**. Scientific Reports 2014, **4**(1):5923.

40. Suzuki H, Ahn HW, Chu T, Bowden W, Gassei K, Orwig K, Rajkovic A: **SOHLH1 and SOHLH2 coordinate spermatogonial differentiation**. Developmental Biology 2012, **361**(2):301-312.

41. Garbuzov A, Pech MF, Hasegawa K, Sukhwani M, Zhang RJ, Orwig KE, Artandi SE: **Purification of GFRalpha1+ and GFRalpha1- spermatogonial stem cells reveals a niche-dependent mechanism for fate determination**. Stem Cell Reports 2018, **10**(2):553-567.

42. Gassei K, Orwig KE: **SALL4 expression in gonocytes and spermatogonial clones of postnatal mouse testes**. PLoS One 2013, **8**(1):e53976.

43. Ebata KT, Zhang X, Nagano MC: **Expression patterns of cell-surface molecules on male germ line stem cells during postnatal mouse development**. Molecular Reproduction and Development 2005, **72**(2):171-181.

44. Zhang T, Oatley J, Bardwell VJ, Zarkower D: **DMRT1 Is required for mouse spermatogonial stem cell maintenance and replenishment**. PLOS Genetics 2016, **12**(9):e1006293.

45. La HM, Chan AL, Legrand JMD, Rossello FJ, Gangemi CG, Papa A, Cheng Q, Morand EF, Hobbs RM: **GILZ-dependent modulation of mTORC1 regulates spermatogonial maintenance**. Development 2018, **145**(18).

46. Tokue M, Ikami K, Mizuno S, Takagi C, Miyagi A, Takada R, Noda C, Kitadate Y, Hara K, Mizuguchi H et al: **SHISA6 confers resistance to differentiation-promoting Wnt/β-Catenin signaling in mouse spermatogenic stem cells**. Stem Cell Reports 2017, **8**(3):561-575.

47. Nakagawa T, Sharma M, Nabeshima Y, Braun RE, Yoshida S: **Functional hierarchy and reversibility within the murine spermatogenic stem cell compartment**. Science 2010, **328**(5974):62-67.

48. Du G, Wang X, Luo M, Xu W, Zhou T, Wang M, Yu L, Li L, Cai Le, Wang PJ et al: **mRBPome capture identifies the RNA-binding protein TRIM71, an essential regulator of spermatogonial differentiation**. Development 2020, **147**(8):dev184655.

49. Suzuki H, Sada A, Yoshida S, Saga Y: **The heterogeneity of spermatogonia is revealed by their topology and expression of marker proteins including the germ cell-specific proteins Nanos2 and Nanos3**. Developmental Biology 2009, **336**(2):222-231.

50. Sada A, Suzuki A, Suzuki H, Saga Y: **The RNA-binding protein NANOS2 is required to maintain murine spermatogonial stem cells**. Science 2009, **325**(5946):1394-1398.

51. Goertz MJ, Wu Z, Gallardo TD, Hamra FK, Castrillon DH: **Foxo1 is required in mouse spermatogonial stem cells for their maintenance and the initiation of spermatogenesis**. The Journal of Clinical Investigation 2011, **121**(9):3456-3466.

52. Takase HM, Nusse R: **Paracrine Wnt/β-catenin signaling mediates proliferation of undifferentiated spermatogonia in the adult mouse testis**. Proceedings of the National Academy of Sciences 2016:201601461.

53. McAninch D, Mäkelä J-A, La HM, Hughes JN, Lovell-Badge R, Hobbs RM, Thomas PQ: **SOX3 promotes generation of committed spermatogonia in postnatal mouse testes**. Scientific Reports 2020, **10**(1):6751.

54. Nakamura Y, Jörg DJ, Kon Y, Simons BD, Yoshida S: **Transient suppression of transplanted spermatogonial stem cell differentiation restores fertility in mice**. Cell Stem Cell 2021.

55. Hara K, Nakagawa T, Enomoto H, Suzuki M, Yamamoto M, Simons Benjamin D, Yoshida S: **Mouse Spermatogenic Stem Cells Continually Interconvert between Equipotent Singly Isolated and Syncytial States**. Cell Stem Cell 2014, **14**(5):658-672.

56. Campos-Junior PHA, Costa GMJ, Lacerda SMSN, Rezende-Neto JV, de Paula AM, Hofmann M-C, de França LR: **The Spermatogonial Stem Cell Niche in the Collared Peccary (Tayassu tajacu)1**. Biology of Reproduction 2012, **86**(5).

57. Lara NdLeM, Costa GMJ, Avelar GF, Guimarães DA, França LR: **Postnatal testis development in the collared peccary (Tayassu tajacu), with emphasis on spermatogonial stem cells markers and niche**. General and Comparative Endocrinology 2019, **273**:98-107.

58. Lee KH, Lee WY, Do JT, Park CK, Kim NH, Kim JH, Chung HJ, Kim DW, Song H: **In vitro ectopic behavior of porcine spermatogonial germ cells and testicular somatic cells**. Cellular Reprogramming 2016, **18**(4):246-255.

59. Lee KH, Lee WY, Kim JH, Yoon MJ, Kim NH, Uhm SJ, Kim DH, Chung HJ, Song H: **Characterization of GFRalpha-1-positive and GFRalpha-1-negative spermatogonia in neonatal pig testis**. Reproduction in Domestic Animals 2013, **48**(6):954-960.

60. Liu T, Zhang P, Li T, Chen X, Zhu Z, Lyu Y, Li X, Tian X, Zeng W: **SETDB1 plays an essential role in maintenance of gonocyte survival in pigs**. Reproduction 2017, **154**(1):23.

61. Gassei K, Ehmcke J, Schlatt S: **Efficient enrichment of undifferentiated GFR alpha 1+ spermatogonia from immature rat testis by magnetic activated cell sorting**. Cell and Tissue Research 2009, **337**(1):177-183.

62. Zhang Y, Su H, Luo F, Wu S, Liu L, Liu T, Yu B, Wu Y: **E-cadherin can be expressed by a small population of rat undifferentiated spermatogonia in vivo and in vitro**. In Vitro Cellular & Developmental Biology - Animal 2011, **47**(8):593-600.

63. Sahare M, Kim S-M, Otomo A, Komatsu K, Minami N, Yamada M, Imai H: **Factors supporting long-term culture of bovine male germ cells**. Reproduction, Fertility and Development 2016, **28**(12):2039-2050.

64. Kim K-J, Lee Y-A, Kim B-J, Kim Y-H, Kim B-G, Kang H-G, Jung S-E, Choi S-H, Schmidt JA, Ryu B-Y: **Cryopreservation of putative pre-pubertal bovine spermatogonial stem cells by slow freezing**. Cryobiology 2015, **70**(2):175-183.

65. Suyatno, Kitamura Y, Ikeda S, Minami N, Yamada M, Imai H: **Long-term culture of undifferentiated spermatogonia isolated from immature and adult bovine testes**. Molecular Reproduction and Development 2018, **85**(3):236-249.

66. Zhu H, Liu C, Sun J, Li M, Hua J: **Effect of GSK-3 inhibitor on the proliferation of multipotent male germ line stem cells (mGSCs) derived from goat testis**. Theriogenology 2012, **77**(9):1939-1950.

67. Song W, Zhu H, Li M, Li N, Wu J, Mu H, Yao X, Han W, Liu W, Hua J: **Promyelocytic leukaemia zinc finger maintains self-renewal of male germline stem cells (mGSCs) and its expression pattern in dairy goat testis**. Cell Proliferation 2013, **46**(4):457-468.

68. Lim JJ, Kim HJ, Kim KS, Hong JY, Lee DR: **In vitro culture-induced pluripotency of human spermatogonial stem cells**. BioMed Research International 2013, **2013**:143028.

69. Chen Z, Sun M, Yuan Q, Niu M, Yao C, Hou J, Wang H, Wen L, Liu Y, Li Z et al: **Generation of functional hepatocytes from human spermatogonial stem cells**. Oncotarget 2016, **7**(8):8879-8895.

70. Yang H, Hao D, Liu C, Huang D, Chen B, Fan H, Liu C, Zhang L, Zhang Q, An J et al: **Generation of functional dopaminergic neurons from human spermatogonial stem cells to rescue parkinsonian phenotypes**. Stem Cell Research & Therapy 2019, **10**(1):195.

71. Golestaneh N, Kokkinaki M, Pant D, Jiang J, DeStefano D, Fernandez-Bueno C, Rone JD, Haddad BR, Gallicano GI, Dym M: **Pluripotent stem cells derived from adult human testes**. Stem Cells and Development 2009, **18**(8):1115-1125.

72. Gassei K, Ehmcke J, Dhir R, Schlatt S: **Magnetic activated cell sorting allows isolation of spermatogonia from adult primate testes and reveals distinct GFRa1-positive subpopulations in men**. Journal of Medical Primatology 2010, **39**(2):83-91.

73. Guo Y, Liu L, Sun M, Hai Y, Li Z, He Z: **Expansion and long-term culture of human spermatogonial stem cells via the activation of SMAD3 and AKT pathways**. Experimental Biology and Medicine 2015, **240**(8):1112-1122.

74. Karmakar PC, Kang H-G, Kim Y-H, Jung S-E, Rahman MS, Lee H-S, Kim Y-H, Pang M-G, Ryu B-Y: **Bisphenol A affects on the functional properties and proteome of testicular germ cells and spermatogonial stem cells in vitro culture model**. Scientific Reports 2017, **7**(1):11858.

75. Zhou Q, Guo Y, Zheng B, Shao B, Jiang M, Wang G, Zhou T, Wang L, Zhou Z, Guo X et al: **Establishment of a proteome profile and identification of molecular markers for mouse spermatogonial stem cells**. Journal of Cellular and Molecular Medicine 2015, **19**(3):521-534.

76. Wang S, Wang X, Wu Y, Han C: **IGF-1R signaling is essential for the proliferation of cultured mouse spermatogonial stem cells by promoting the G2/M progression of the cell cycle**. Stem Cells Development 2015, **24**(4):471-483.

77. Kokkinaki M, Lee T-L, He Z, Jiang J, Golestaneh N, Hofmann M-C, Chan W-Y, Dym M: **The molecular signature of spermatogonial stem/progenitor cells in the 6-day-old mouse testis**. Biology of Reproduction 2009, **80**(4):707-717.

78. Lord T, Oatley MJ, Oatley JM: **Testicular architecture is critical for mediation of retinoic acid responsiveness by undifferentiated spermatogonial subtypes in the mouse**. Stem Cell Reports 2018, **10**(2):538-552.

79. Lee YA, Kim YH, Ha SJ, Kim BJ, Kim KJ, Jung MS, Kim BG, Ryu BY: **Effect of sugar molecules on the cryopreservation of mouse spermatogonial stem cells**. Fertility and Sterility 2014, **101**(4):1165-1175 e1165.

80. Zheng K, Wu X, Kaestner KH, Wang PJ: **The pluripotency factor LIN28 marks undifferentiated spermatogonia in mouse**. BMC Developmental Biology 2009, **9**:38.

81. Chan AL, La HM, Legrand JMD, Makela JA, Eichenlaub M, De Seram M, Ramialison M, Hobbs RM: **Germline stem cell activity is sustained by SALL4-dependent silencing of distinct tumor suppressor genes**. Stem Cell Reports 2017, **9**(3):956-971.

82. Wang J, Li J, Xu W, Xia Q, Gu Y, Song W, Zhang X, Yang Y, Wang W, Li H et al: **Androgen promotes differentiation of PLZF(+) spermatogonia pool via indirect regulatory pattern**. Cell Communication and Signaling 2019, **17**(1):57.

83. Yu K, Zhang Y, Zhang B-L, Wu H-Y, Jiang W-Q, Wang S-T, Han D-P, Liu Y-X, Lian Z-X, Deng S-L: **In-vitro differentiation of early pig spermatogenic cells to haploid germ cells**. Molecular Human Reproduction 2019, **25**(9):507-518.

84. Lee KH, Lee WY, Kim JH, Park CK, Do JT, Kim JH, Choi YS, Kim NH, Song H: **Subculture of germ cell-derived colonies with GATA4-positive feeder cells from neonatal pig testes**. Stem Cells International 2016, **2016**:9.

85. Zhang P, Chen X, Zheng Y, Zhu J, Qin Y, Lv Y, Zeng W: **Long-term propagation of porcine undifferentiated spermatogonia**. Stem Cells and Development 2017, **26**(15):1121-1131.

86. Zhang P, Qin Y, Zheng Y, Zeng W: **Phospholipase D family member 6 is a surface marker for enrichment of undifferentiated spermatogonia in prepubertal boars**. Stem Cells and Development 2017, **27**(1):55-64.

87. Kubota H, Wu X, Goodyear SM, Avarbock MR, Brinster RL: **Glial cell line-derived neurotrophic factor and endothelial cells promote self-renewal of rabbit germ cells with spermatogonial stem cell properties**. The FASEB Journal 2011, **25**(8):2604-2614.

88. Wang M, Zhang C, Huang C, Cheng S, He N, Wang Y, Ahmed MF, Zhao R, Jin J, Zuo Q et al: **Regulation of fibroblast growth factor 8 (FGF8) in chicken embryonic stem cells differentiation into spermatogonial stem cells**. Journal of Cellular Biochemistry 2018, **119**(2):2396-2407.

89. Zhang C, Wang F, Zuo Q, Sun C, Jin J, Li T, Wang M, Zhao R, Yu X, Sun H et al: **Cped1 promotes chicken SSCs formation with the aid of histone acetylation and transcription factor Sox2**. Bioscience Reports 2018, **38**(5).

90. Wang Y, Bi Y, Zuo Q, Zhang W, Li D, He N-n, Cheng S, Zhang Y-n, Li B: **MAPK8 regulates chicken male germ cell differentiation through JNK signaling pathway**. Journal of Cellular Biochemistry 2018, **119**(2):1548-1557.

91. Zhang C, Wang M, He N, Ahmed MF, Wang Y, Zhao R, Yu X, Jin J, Song J, Zuo Q et al: **Hsd3b2 associated in modulating steroid hormone synthesis pathway regulates the differentiation of chicken embryonic stem cells into spermatogonial stem cells**. Journal of Cellular Biochemistry 2018, **119**(1):1111-1121.

92. Wang Y, Zuo Q, Bi Y, Zhang W, Jin J, Zhang L, Zhang YN, Li B: **miR-31 regulates spermatogonial stem cells meiosis via targeting Stra8**. Journal of Cellular Biochemistry 2017, **118**(12):4844-4853.

93. Zuo Q, Zhang C, Jin K, Jing J, Sun C, Ahmed MF, Song J, Zhang Y, Chen G, Li B: **NICD-mediated notch transduction regulates the different fate of chicken primordial germ cells and spermatogonial stem cells**. Cell & Bioscience 2018, **8**:40-40.

94. Zhang W, Bi Y, Wang Y, Wang M, Li D, Cheng S, Jin J, Li T, Li B, Zhang Y: **Nanos2 promotes differentiation of male germ cells basing on the negative regulation of Foxd3 and the treatment of 5-Azadc and TSA**. Journal of Cellular Physiology 2019, **234**(4):3762-3774.

95. Zhang W, Bi Y, Wang Y, Li D, He N, Wang M, Jin J, Zuo Q, Zhang Y: **Nanos2 promotes differentiation of chicken (Gallus gallus) embryonic stem cells to male germ cells**. Journal of Cellular Biochemistry 2018, **119**(6):4435-4446.

96. He N, Wang Y, Zhang C, Wang M, Wang Y, Zuo Q, Zhang Y, Li B: **Wnt signaling pathway regulates differentiation of chicken embryonic stem cells into spermatogonial stem cells via Wnt5a**. Journal of Cellular Biochemistry 2018, **119**(2):1689-1701.

97. Kong L, Qiu L, Guo Q, Chen Y, Zhang X, Chen B, Zhang Y, Chang G: **Long-term in vitro culture and preliminary establishment of chicken primordial germ cell lines**. PLoS One 2018, **13**(4):e0196459.

98. Zhang W, Bi Y, Wang Y, Wang M, Li D, Cheng S, Jin J, Li T, Li B, Zhang Y: **Nanos2 promotes differentiation of male germ cells basing on the negative regulation of Foxd3 and the treatment of 5-Azadc and TSA**. Journal of Cellular Physiology 2019, **234**(4):3762-3774.

99. Izadyar F, Wong J, Maki C, Pacchiarotti J, Ramos T, Howerton K, Yuen C, Greilach S, Zhao HH, Chow M et al: **Identification and characterization of repopulating spermatogonial stem cells from the adult human testis**. Human Reproduction 2011, **26**(6):1296-1306.

100. Nasiri Z, Hosseini SM, Hajian M, Abedi P, Bahadorani M, Baharvand H, Nasr-Esfahani MH: **Effects of different feeder layers on short-term culture of prepubertal bovine testicular germ cells In-vitro**. Theriogenology 2012, **77**(8):1519-1528.

101. Bahadorani M, Hosseini SM, Abedi P, Abbasi H, Nasr-Esfahani MH: **Glial cell line-derived neurotrophic factor in combination with insulin-like growth factor 1 and basic fibroblast growth factor promote in vitro culture of goat spermatogonial stem cells**. Growth Factors 2015, **33**(3):181-191.

102. Pramod RK, Mitra A: **In vitro culture and characterization of spermatogonial stem cells on Sertoli cell feeder layer in goat (Capra hircus)**. Journal of Assisted Reproduction and Genetics 2014, **31**(8):993-1001.

103. Wang J, Cao H, Xue X, Fan C, Fang F, Zhou J, Zhang Y, Zhang X: **Effect of vitamin C on growth of caprine spermatogonial stem cells in vitro**. Theriogenology 2014, **81**(4):545-555.

104. Bahadorani M, Hosseini SM, Abedi P, Hajian M, Hosseini SE, Vahdati A, Baharvand H, Nasr-Esfahani MH: **Short-term in-vitro culture of goat enriched spermatogonial stem cells using different serum concentrations**. Journal of Assisted Reproduction and Genetics 2012, **29**(1):39-46.

105. Gholami K, Pourmand G, Koruji M, Sadighigilani M, Navid S, Izadyar F, Abbasi M: **Efficiency of colony formation and differentiation of human spermatogenic cells in two different culture systems**. Reproductive Biology 2018, **18**(4):397-403.

106. Conrad S, Renninger M, Hennenlotter J, Wiesner T, Just L, Bonin M, Aicher W, Bühring H-J, Mattheus U, Mack A et al: **Generation of pluripotent stem cells from adult human testis**. Nature 2008, **456**(7220):344-349.

107. Shinohara T, Avarbock MR, Brinster RL: **b1- and a6-integrin are surface markers on mouse spermatogonial stem cells**. Proceedings of the National Academy of Sciences of the United States of America 1999, **96**(10):5504-5509.

108. Shiura H, Ikeda R, Lee J, Sato T, Ogonuki N, Hirose M, Ogura A, Ogawa T, Abe K: **Generation of a novel germline stem cell line expressing a germline-specific reporter in the mouse**. Genesis 2013, **51**(7):498-505.

109. Shinohara T, Orwig KE, Avarbock MR, Brinster RL: **Spermatogonial stem cell enrichment by multiparameter selection of mouse testis cells**. Proceedings of the National Academy of Sciences of the United States of America 2000, **97**(15):8346-8351.

110. Kanatsu-Shinohara M, Miki H, Inoue K, Ogonuki N, Toyokuni S, Ogura A, Shinohara T: **Long-term culture of mouse male germline stem cells under serum-or feeder-free conditions**. Biology of Reproduction 2005, **72**(4):985-991.

111. Kanatsu-Shinohara M, Inoue K, Takashima S, Takehashi M, Ogonuki N, Morimoto H, Nagasawa T, Ogura A, Shinohara T: **Reconstitution of mouse spermatogonial stem cell niches in culture**. Cell Stem Cell 2012, **11**(4):567-578.

112. Kim YH, Kim BJ, Kim BG, Lee YA, Kim KJ, Chung HJ, Hwang S, Woo JS, Park JK, Schmidt JA et al: **Stage-specific embryonic antigen-1 expression by undifferentiated spermatogonia in the prepubertal boar testis**. Journal of Animal Science 2013, **91**(7):3143-3154.

113. Jin J, Zhao R: **The Lbc gene promotes differentiation of chicken embryo stem cell into spermatogonial stem cells via the regulation of transcriptional factor Hoxa5**. Journal of Cellular Biochemistry 2019.

114. Shi R, Bai Y, Li S, Wei H, Zhang X, Li L, Tian XC, Jiang Q, Wang C, Qin L et al: **Characteristics of spermatogonial stem cells derived from neonatal porcine testis**. Andrologia 2015, **47**(7):765-778.

115. Panda RP, Barman HK, Mohapatra C: **Isolation of enriched carp spermatogonial stem cells from Labeo rohita testis for in vitro propagation**. Theriogenology 2011, **76**(2):241-251.

116. Nayak S, Ferosekhan S, Sahoo SK, Sundaray JK, Jayasankar P, Barman HK: **Production of fertile sperm from in vitro propagating enriched spermatogonial stem cells of farmed catfish, Clarias batrachus**. Zygote 2016, **24**(6):814-824.

117. Shi L, Zhao H, Ren Y, Yao X, Song R, Yue W: **Effects of different levels of dietary selenium on the proliferation of spermatogonial stem cells and antioxidant status in testis of roosters**. Animal Reproduction Science 2014, **149**(3-4):266-272.

118. Feng W, Chen S, Do D, Liu Q, Deng Y, Lei X, Luo C, Huang B, Shi D: **Isolation and identification of prepubertal buffalo (Bubalus bubalis) spermatogonial stem cells**. Asian-Australasian Journal of Animal Sciences 2016, **29**(10):1407-1415.

119. Reding SC, Stepnoski AL, Cloninger EW, Oatley JM: **THY1 is a conserved marker of undifferentiated spermatogonia in the pre-pubertal bull testis**. Reproduction 2010, **139**(5):893.

120. Abbasi H, Tahmoorespur M, Hosseini SM, Nasiri Z, Bahadorani M, Hajian M, Nasiri MR, Nasr-Esfahani MH: **THY1 as a reliable marker for enrichment of undifferentiated spermatogonia in the goat**. Theriogenology 2013, **80**(8):923-932.

121. Smith JF, Yango P, Altman E, Choudhry S, Poelzl A, Zamah AM, Rosen M, Klatsky PC, Tran ND: **Testicular niche required for human spermatogonial stem cell expansion**. STEM CELLS Translational Medicine 2014, **3**(9):1043-1054.

122. Altman E, Yango P, Moustafa R, Smith JF, Klatsky PC, Tran ND: **Characterization of human spermatogonial stem cell markers in fetal, pediatric, and adult testicular tissues**. Reproduction 2014, **148**(4):417.

123. Li C-H, Yan L-Z, Ban W-Z, Tu Q, Wu Y, Wang L, Bi R, Ji S, Ma Y-H, Nie W-H et al: **Long-term propagation of tree shrew spermatogonial stem cells in culture and successful generation of transgenic offspring**. Cell Research 2017, **27**(2):241-252.

124. Sharma A, Shah SM, Tiwari M, Roshan M, Singh MK, Singla SK, Palta P, Manik RS, Chauhan MS: **Propagation of goat putative spermatogonial stem cells under growth factors defined serum-free culture conditions**. Cytotechnology 2020(72):489–497.

125. Hermann BP, Sukhwani M, Winkler F, Pascarella JN, Peters KA, Sheng Y, Valli H, Rodriguez M, Ezzelarab M, Dargo G et al: **Spermatogonial stem cell transplantation into rhesus testes regenerates spermatogenesis producing functional sperm**. Cell Stem Cell 2012, **11**(5):715-726.

126. Filipponi D, Hobbs RM, Ottolenghi S, Rossi P, Jannini EA, Pandolfi PP, Dolci S: **Repression of kit expression by Plzf in germ cells**. Molecular and Cellular Biology 2007, **27**(19):6770-6781.

127. Azizi H, Ranjbar M, Rahaiee S, Govahi M, Skutella T: **Investigation of VASA gene and protein expression in neonate and adult testicular germ cells in mice in vivo and in vitro**. Cell journal 2020, **22**(2):171-177.

128. Zhao H, Nie J, Zhu X, Lu Y, Liang X, Xu H, Yang X, Zhang Y, Lu K, Lu S: **In vitro differentiation of spermatogonial stem cells using testicular cells from Guangxi Bama mini-pig**. Journal of Veterinary Science 2018, **19**(5):592-599.

129. Jung JG, Lee YM, Kim JN, Kim TM, Shin JH, Kim TH, Lim JM, Han JY: **The reversible developmental unipotency of germ cells in chicken**. Reproduction 2010, **139**(1):113.

130. Lavial F, Acloque H, Bachelard E, Nieto MA, Samarut J, Pain B: **Ectopic expression of Cvh (Chicken Vasa homologue) mediates the reprogramming of chicken embryonic stem cells to a germ cell fate**. Developmental Biology 2009, **330**(1):73-82.

131. Li B, Wang XY, Tian Z, Xiao XJ, Xu Q, Wei CX, F.Y, Sun HC, Chen GH: **Directional differentiation of chicken spermatogonial stem cells in vitro**. Cytotherapy 2010, **12**(3):326-331.

132. Yu F, Ding L-J, Sun G-B, Sun P-X, He X-H, Ni L-G, Li B-C: **Transgenic sperm produced by electrotransfection and allogeneic transplantation of chicken fetal spermatogonial stem cells**. Molecular Reproduction and Development 2010, **77**(4):340-347.

133. Lee YM, Jung JG, Kim JN, Park TS, Kim TM, Shin SS, Kang DK, Lim JM, Han JY: **A testis-mediated germline chimera production based on transfer of chicken testicular cells directly into heterologous testes**. Biology of Reproduction 2006, **75**(3):380-386.

134. Goel S, Reddy N, Mahla RS, Suman SK, Pawar RM: **Spermatogonial stem cells in the testis of an endangered bovid: Indian black buck (Antilope cervicapra L.)**. Animal Reproduction Science 2011, **126**(3):251-257.

135. Powell RH, Galiguis J, Biancardi MN, Pope CE, Leibo SP, Wang G, Gómez MC: **Phenotypic and molecular characterization of domestic cat (Felis catus) spermatogonial stem cells**. Biology of Reproduction 2016, **95**(1):1-10.

136. Guan K, Nayernia K, Maier LS, Wagner S, Dressel R, Lee JH, Nolte J, Wolf F, Li M, Engel W et al: **Pluripotency of spermatogonial stem cells from adult mouse testis**. Nature 2006, **440**(7088):1199-1203.

137. Pandey V, Tripathi A, Dubey PK: **Expression and intracellular localization of Nanos2-homologue protein in primordial germ cells and spermatogonial stem cells**. Zygote 2019, **27**(2):82-88.

138. Hickford DE, Frankenberg S, Pask AJ, Shaw G, Renfree MB: **DDX4 (VASA) is conserved in germ cell development in marsupials and monotremes**. Biology of Reproduction 2011, **85**(4):733-743.

139. Wang X, Chen T, Zhang Y, Li B, Xu Q, Song C: **Isolation and culture of pig spermatogonial stem cells and their in vitro differentiation into neuron-like cells and adipocytes**. International Journal of Molecular Sciences 2015, **16**(11):26333-26346.

140. Muller T, Eildermann K, Dhir R, Schlatt S, Behr R: **Glycan stem-cell markers are specifically expressed by spermatogonia in the adult non-human primate testis**. Human Reproduction 2008, **23**(10):2292-2298.

141. Kokkinaki M, Djourabtchi A, Golestaneh N: **Long-term culture of human SSEA-4 positive spermatogonial stem cells (SSCs)**. Journal of Stem Cell Research & Therapy 2011, **2**(2).

142. Liu S, Tang Z, Xiong T, Tang W: **Isolation and characterization of human spermatogonial stem cells**. Reproductive Biology and Endocrinology 2011, **9**(1):141.

143. Mohapatra C, Barman HK: **Identification of promoter within the first intron of Plzf gene expressed in carp spermatogonial stem cells**. Molecular Biology Reports 2014, **41**(10):6433-6440.

144. Lacerda S, Martinez ERM, Mura I, Doretto LB, Costa GMJ, Silva MA, Digmayer M, Nobrega RH, Franca LR: **Duration of spermatogenesis and identification of spermatogonial stem cell markers in a Neotropical catfish, Jundia (Rhamdia quelen)**. General and Comparative Endocrinology 2019, **273**:249-259.

145. Bellaiche J, Lareyre J-J, Cauty C, Yano A, Allemand I, Le Gac F: **Spermatogonial Stem Cell Quest: nanos2, Marker of a Subpopulation of Undifferentiated A Spermatogonia in Trout Testis1**. Biology of Reproduction 2014, **90**(4).

146. Ozaki Y, Saito K, Shinya M, Kawasaki T, Sakai N: **Evaluation of Sycp3, Plzf and Cyclin B3 expression and suitability as spermatogonia and spermatocyte markers in zebrafish**. Gene Expression Patterns 2011, **11**(5):309-315.

147. McMillan M, Andronicos N, Davey R, Stockwell S, Hinch G, Schmoelzl S: **Claudin-8 expression in Sertoli cells and putative spermatogonial stem cells in the bovine testis**. Reproduction, Fertility and Development 2014, **26**(5):633-644.

148. Oatley MJ, Kaucher AV, Yang Q-E, Waqas MS, Oatley JM: **Conditions for long-term culture of cattle undifferentiated spermatogonia**. Biology of Reproduction 2016, **95**(1).

149. Bedford-Guaus S, Kim S, Mulero L, Vaquero J, Morera C, Adan-Milanès R, Veiga A, Raya Á: **Molecular markers of putative spermatogonial stem cells in the domestic cat**. Reproduction in Domestic Animals 2017, **52**(S2):177-186.

150. Harkey MA, Asano A, Zoulas ME, Torok-Storb B, Nagashima J, Travis A: **Isolation, genetic manipulation, and transplantation of canine spermatogonial stem cells: progress toward transgenesis through the male germ-line**. Reproduction 2013, **146**(1):75-90.

151. Mu H, Li N, Wu J, Zheng L, Zhai Y, Li B, Song W, Wang J, Zhu H, Li G et al: **PLZF-induced upregulation of CXCR4 promotes dairy goat male germline stem cell proliferation by targeting mir146a**. Journal of Cellular Biochemistry 2016, **117**(4):844-852.

152. Sadri-Ardekani H, Mizrak SC, van Daalen SKM, Korver CM, Roepers-Gajadien HL, Koruji M, Hovingh S, de Reijke TM, de la Rosette JJMCH, van der Veen F et al: **Propagation of Human Spermatogonial Stem Cells In Vitro**. JAMA 2009, **302**(19):2127-2134.

153. Costoya JA, Hobbs RM, Barna M, Cattoretti G, Manova K, Sukhwani M, Orwig KE, Wolgemuth DJ, Pandolfi PP: **Essential role of Plzf in maintenance of spermatogonial stem cells**. Nature Genetics 2004, **36**(6):653-659.

154. Mohamadi SM, Movahedin M, Koruji SM, Jafarabadi MA, Makoolati Z: **Comparison of colony formation in adult mouse spermatogonial stem cells developed in Sertoli and STO coculture systems**. Andrologia 2012, **44**(s1):431-437.

155. Yang QE, Kim D, Kaucher A, Oatley MJ, Oatley JM: **CXCL12-CXCR4 signaling is required for the maintenance of mouse spermatogonial stem cells**. Journal of Cell Science 2013, **126**(Pt 4):1009-1020.

156. Pech MF, Garbuzov A, Hasegawa K, Sukhwani M, Zhang RJ, Benayoun BA, Brockman SA, Lin S, Brunet A, Orwig KE et al: **High telomerase is a hallmark of undifferentiated spermatogonia and is required for maintenance of male germline stem cells**. Genes & Development 2015, **29**(23):2420-2434.

157. Li H, Liang Z, Yang J, Wang D, Wang H, Zhu M, Geng B, Xu EY: **DAZL is a master translational regulator of murine spermatogenesis**. National Science Review 2018, **6**(3):455-468.

158. Legrand JMD, Chan A-L, La HM, Rossello FJ, Änkö M-L, Fuller-Pace FV, Hobbs RM: **DDX5 plays essential transcriptional and post-transcriptional roles in the maintenance and function of spermatogonia**. Nature Communications 2019, **10**(1):2278.

159. Iwamori N, Iwamori T, Matzuk MM: **H3K27 demethylase, JMJD3, regulates fragmentation of spermatogonial cysts**. PLoS One 2013, **8**(8):e72689.

160. Zheng B, Zhou Q, Guo Y, Shao B, Zhou T, Wang L, Zhou Z, Sha J, Guo X, Huang X: **Establishment of a proteomic profile associated with gonocyte and spermatogonial stem cell maturation and differentiation in neonatal mice**. Proteomics 2014, **14**(2-3):274-285.

161. Costoya JA, Hobbs RM, Barna M, Cattoretti G, Manova K, Sukhwani M, Orwig KE, Wolgemuth DJ, Pandolfi PP: **Essential role of Plzf in maintenance of spermatogonial stem cells**. Nature Genetics 2004, **36**(6):653-659.

162. Hobbs RM, Seandel M, Falciatori I, Rafii S, Pandolfi PP: **Plzf regulates germline progenitor self-renewal by opposing mTORC1**. Cell 2010, **142**(3):468-479.

163. Bhang DH, Kim B-J, Kim BG, Schadler K, Baek K-H, Kim YH, Hsiao W, Ding B-S, Rafii S, Weiss MJ et al: **Testicular endothelial cells are a critical population in the germline stem cell niche**. Nature Communications 2018, **9**(1):4379.

164. Tahara N, Kawakami H, Zhang T, Zarkower D, Kawakami Y: **Temporal changes of Sall4 lineage contribution in developing embryos and the contribution of Sall4-lineages to postnatal germ cells in mice**. Scientific Reports 2018, **8**(1):16410.

165. Bordlein A, Scherthan H, Nelkenbrecher C, Molter T, Bosl MR, Dippold C, Birke K, Kinkley S, Staege H, Will H et al: **SPOC1 (PHF13) is required for spermatogonial stem cell differentiation and sustained spermatogenesis**. Journal of Cell Science 2011, **124**(Pt 18):3137-3148.

166. Ballow D, Meistrich ML, Matzuk M, Rajkovic A: **Sohlh1 is essential for spermatogonial differentiation**. Developmental Biology 2006, **294**(1):161-167.

167. Yang Q-E, Gwost I, Oatley MJ, Oatley JM: **Retinoblastoma protein (RB1) controls fate determination in stem cells and progenitors of the mouse male germline**. Biology of Reproduction 2013, **89**(5).

168. Liao J, Ng SH, Tu J, Shui Luk AC, Qian Y, Fung J, Sang Tang NL, Feng B, Chan W-Y, Fouchet P et al: **Single-cell RNA-Seq resolves cellular heterogeneity and transcriptional dynamics in spermatogonial stem cells establishment**. bioRxiv 2018:194696.

169. Buaas FW, Kirsh AL, Sharma M, McLean DJ, Morris JL, Griswold MD, de Rooij DG, Braun RE: **Plzf is required in adult male germ cells for stem cell self-renewal**. Nature Genetics 2004, **36**(6):647-652.

170. Helsel AR, Yang Q-E, Oatley MJ, Lord T, Sablitzky F, Oatley JM: **ID4 levels dictate the stem cell state in mouse spermatogonia**. Development 2017, **144**(4):624-634.

171. Qi L, Li J, Le W, Zhang J: **Low-dose ionizing irradiation triggers apoptosis of undifferentiated spermatogonia in vivo and in vitro**. Translational Andrology and Urology 2019, **8**(6):591-600.

172. Choi YH, Park C-H, Kim W, Ling H, Kang A, Chang MW, Im S-K, Jeong H-W, Kong Y-Y, Kim K-T: **Vaccinia-related kinase 1 Is required for the maintenance of undifferentiated spermatogonia in mouse male germ cells**. PLoS One 2010, **5**(12):e15254.

173. Chen Y, Ma L, Hogarth C, Wei G, Griswold MD, Tong M-H: **Retinoid signaling controls spermatogonial differentiation by regulating expression of replication-dependent core histone genes**. Development 2016, **143**(9):1502-1511.

174. Payne CJ, Gallagher SJ, Foreman O, Dannenberg JH, Depinho RA, Braun RE: **Sin3a is required by Sertoli cells to establish a niche for undifferentiated spermatogonia, germ cell tumors, and spermatid elongation**. Stem Cells 2010, **28**(8):1424-1434.

175. Wei Y, Yang D, Du X, Yu X, Zhang M, Tang F, Ma F, Li N, Bai C, Li G et al: **Interaction between DMRT1 and PLZF protein regulates self-renewal and proliferation in male germline stem cells**. Molecular and Cellular Biochemistry 2021, **476**(2):1123-1134.

176. Lee YA, Kim YH, Ha SJ, Kim KJ, Kim BJ, Kim BG, Choi SH, Kim IC, Schmidt JA, Ryu BY: **Cryopreservation of porcine spermatogonial stem cells by slow-freezing testis tissue in trehalose1**. Journal of Animal Science 2014, **92**(3):984-995.

177. Luo J, Megee S, Dobrinski I: **Asymmetric Distribution of UCH-L1 in Spermatogonia Is Associated With Maintenance and Differentiation of Spermatogonial Stem Cells**. Journal of Cellular Physiology 2009, **220**(2):460-468.

178. Almunia J, Nakamura K, Murakami M, Takashima S, Mori T, Takasu M: **Sexual precocity in male microminipigs evaluated immunohistologically using spermatogonial stem cell markers**. Theriogenology 2019, **130**:120-124.

179. Alves-Lopes JP, Soder O, Stukenborg JB: **Testicular organoid generation by a novel in vitro three-layer gradient system**. Biomaterials 2017, **130**:76-89.

180. Zhang Y, Wu S, Luo F-h, Baiyinbatu, Liu L-h, Hu T-y, Yu B, Li G-p, Wu Y-j: **CDH1, a novel surface marker of spermatogonial stem cells in sheep testis**. Journal of Integrative Agriculture 2014, **13**(8):1759-1765.

181. Wang G, Li Y, Yang Q, Xu S, Ma S, Yan R, Zhang R, Jia G, Ai D, Yang Qe: **Gene expression dynamics during the gonocyte to spermatogonia transition and spermatogenesis in the domestic yak**. Journal of Animal Science and Biotechnology 2019, **10**(1):64.

182. Sachs C, Robinson BD, Andres Martin L, Webster T, Gilbert M, Lo HY, Rafii S, Ng CK, Seandel M: **Evaluation of candidate spermatogonial markers ID4 and GPR125 in testes of adult human cadaveric organ donors**. Andrology 2014, **2**(4):607-614.

183. Li Y, Zhang Y, Zhang X, Sun J, Hao J: **BMP4/Smad signaling pathway induces the differentiation of mouse spermatogonial stem cells via upregulation of Sohlh2**. The Anatomical Record 2014, **297**(4):749-757.

184. Li L, Wang M, Wang M, Wu X, Geng L, Xue Y, Wei X, Jia Y, Wu X: **A long non-coding RNA interacts with Gfra1 and maintains survival of mouse spermatogonial stem cells**. Cell Death & Disease 2016, **7**:e2140.

185. Zheng Y, Lei Q, Jongejan A, Mulder CL, van Daalen SKM, Mastenbroek S, Hwang G, Jordan PW, Repping S, Hamer G: **The influence of retinoic acid-induced differentiation on the radiation response of male germline stem cells**. DNA Repair 2018, **70**:55-66.

186. Seandel M, James D, Shmelkov SV, Falciatori I, Kim J, Chavala S, Scherr DS, Zhang F, Torres R, Gale NW et al: **Generation of functional multipotent adult stem cells from GPR125+ germline progenitors**. Nature 2007, **449**(7160):346-350.

187. Youn H, Kim SH, Choi KA, Kim S: **Characterization of Oct4-GFP spermatogonial stem cell line and its application in the reprogramming studies**. Journal of Cellular Biochemistry 2013, **114**(4):920-928.

188. Luo J, Megee S, Rathi R, Dobrinski I: **Protein gene product 9.5 is a spermatogonia-specific marker in the pig testis: Application to enrichment and culture of porcine spermatogonia**. Molecular Reproduction and Development 2006, **73**(12):1531-1540.

189. Kim Y-H, Oh M-G, Bhang DH, Kim B-J, Jung S-E, Kim S-M, Dohr G, Kim S-U, Ryeom S, Ryu B-Y: **Testicular endothelial cells promote self-renewal of spermatogonial stem cells in rats**.Biology of Reproduction 2019, **101**(2):360-367.

190. Wu Z, Falciatori I, Molyneux LA, Richardson TE, Chapman KM, Hamra FK: **Spermatogonial culture medium: an effective and efficient nutrient mixture for culturing rat spermatogonial stem cells**. Biology of Reproduction 2009, **81**(1):77-86.

191. Qasemi-Panahi B, Movahedin M, Moghaddam G, Tajik P, Koruji M, Ashrafi-Helan J, Rafat SA: **Isolation and proliferation of spermatogonial cells from Ghezel sheep**. Avicenna Journal of Medical Biotechnology 2018, **10**(2):93-97.

192. Binsila KB, Selvaraju S, Ghosh SK, Parthipan S, Archana SS, Arangasamy A, Prasad JK, Bhatta R, Ravindra JP: **Isolation and enrichment of putative spermatogonial stem cells from ram (Ovis aries) testis**. Animal Reproduction Science 2018, **196**:9-18.

193. Yang Y, Liu Q, Ma D, Xiao Y, Xu S, Wang X, Song Z, You F, Li J: **Spermatogonial stem cells differentiation and testicular lobules formation in a seasonal breeding teleost: The evidence from the heat-induced masculinization of genetically female Japanese flounder (Paralichthys olivaceus)**. Theriogenology 2018, **120**:68-78.

194. Sánchez-Sánchez AV, Camp E, García-España A, Leal-Tassias A, Mullor JL: **Medaka Oct4 is expressed during early embryo development, and in primordial germ cells and adult gonads**. Developmental Dynamics 2010, **239**(2):672-679.

195. Draper BW: **Identification of germ-line stem cells in zebrafish**. Methods in Molecular Biology (Clifton, NJ) 2017, **1463**:103-113.

196. Yao X, Tang F, Yu M, Zhu H, Chu Z, Li M, Liu W, Hua J, Peng S: **Expression profile of Nanos2 gene in dairy goat and its inhibitory effect on Stra8 during meiosis**. Cell Proliferation 2014, **47**(5):396-405.

197. Mitchell RT, Cowan G, Morris KD, Anderson RA, Fraser HM, McKenzie KJ, Wallace WHB, Kelnar CJH, Saunders PTK, Sharpe RM: **Germ cell differentiation in the marmoset (Callithrix jacchus) during fetal and neonatal life closely parallels that in the human**. Human Reproduction 2008, **23**(12):2755-2765.

198. Tsuda M, Sasaoka Y, Kiso M, Abe K, Haraguchi S, Kobayashi S, Saga Y: **Conserved role of nanos proteins in germ cell development**. Science 2003, **301**(5637):1239.

199. Sisakhtnezhad S, Bahrami AR, Matin MM, Dehghani H, Momeni-Moghaddam M, Boozarpour S, Farshchian M, Dastpak M: **The molecular signature and spermatogenesis potential of newborn chicken spermatogonial stem cells in vitro**. In Vitro Cellular & Developmental Biology - Animal 2015, **51**(4):415-425.

200. Mahla RS, Reddy N, Goel S: **Spermatogonial stem cells (SSCs) in buffalo (Bubalus bubalis) testis**. PLoS One 2012, **7**(4):e36020.

201. Goel S, Reddy N, Mandal S, Fujihara M, Kim S-M, Imai H: **Spermatogonia-specific proteins expressed in prepubertal buffalo (Bubalus bubalis) testis and their utilization for isolation and in vitro cultivation of spermatogonia**. Theriogenology 2010, **74**(7):1221-1232.

202. Vansandt LM, Livesay JL, Dickson MJ, Li L, Pukazhenthi BS, Keefer CL: **Conservation of spermatogonial stem cell marker expression in undifferentiated felid spermatogonia**. Theriogenology 2016, **86**(4):1022-1035.e1023.

203. Vigueras-Villaseñor RM, Cortés-Trujillo L, Chávez-Saldaña M, Vázquez FG, Carrasco-Daza D, Cuevas-Alpuche O, Rojas-Castañeda JC: **Analysis of POU5F1, c-Kit, PLAP, AP2γ and SALL4 in gonocytes of patients with cryptorchidism**. Acta Histochemica 2015, **117**(8):752-761.

204. Clark AT, Gkountela S, Chen D, Liu W, Sosa E, Sukhwani M, Hennebold JD, Orwig KE: **Primate primordial germ cells acquire transplantation potential by carnegie stage 23**. Stem Cell Reports 2017, **9**(1):329-341.

205. Langenstroth D, Kossack N, Westernströer B, Wistuba J, Behr R, Gromoll J, Schlatt S: **Separation of somatic and germ cells is required to establish primate spermatogonial cultures**. Human Reproduction 2014, **29**(9):2018-2031.

206. Zogbi C, Tesser RB, Encinas G, Miraglia SM, Stumpp T: **Gonocyte development in rats: proliferation, distribution and death revisited**. Histochemistry and Cell Biology 2012, **138**(2):305-322.

207. Xu H, Li Z, Li M, Wang L, Hong Y: **Boule is present in fish and bisexually expressed in adult and embryonic germ cells of medaka**. PLoS One 2009, **4**(6):e6097.

208. Kito G, Aramaki S, Tanaka K, Soh T, Yamauchi N, Hattori MA: **Temporal and spatial differential expression of chicken germline-specific proteins cDAZL, CDH and CVH during gametogenesis**. The Journal of Reproduction and Development 2010, **56**(3):341-346.

209. Rengaraj D, Zheng YH, Kang KS, Park KJ, Lee BR, Lee SI, Choi JW, Han JY: **Conserved expression pattern of chicken DAZL in primordial germ cells and germ-line cells**. Theriogenology 2010, **74**(5):765-776.

210. Lee W-Y, Lee R, Park H-J, Do JT, Park C, Kim J-H, Jhun H, Lee J-H, Hur T, Song H: **Characterization of male germ cell markers in canine testis**. Animal Reproduction Science 2017, **182**:1-8.

211. Kim J, Jung H, Yoon M: **VASA (DDX4) is a putative marker for spermatogonia, spermatocytes and round spermatids in stallions**. Reproduction in Domestic Animals 2015, **50**(6):1032-1038.

212. Jung HJ, Song H, Yoon MJ: **Stage-dependent DAZL localization in stallion germ cells**. Animal Reproduction Science 2014, **147**(1):32-38.

213. Anderson RA, Fulton N, Cowan G, Coutts S, Saunders PT: **Conserved and divergent patterns of expression of DAZL, VASA and OCT4 in the germ cells of the human fetal ovary and testis**. BMC Developmental Biology 2007, **7**:136.

214. Yi H, Xiao S, Zhang Y: **Stage-specific approaches promote in vitro induction for spermatogenesis**. In Vitro Cellular & Developmental Biology - Animal 2018, **54**(3):217-230.

215. Yazawa R, Takeuchi Y, Morita T, Ishida M, Yoshizaki G: **The Pacific bluefin tuna (Thunnus orientalis) dead end gene is suitable as a specific molecular marker of type A spermatogonia**. Molecular Reproduction and Development 2013, **80**(10):871-880.

216. Liu L, Hong N, Xu H, Li M, Yan Y, Purwanti Y, Yi M, Li Z, Wang L, Hong Y: **Medaka dead end encodes a cytoplasmic protein and identifies embryonic and adult germ cells**. Gene Expression Patterns 2009, **9**(7):541-548.

217. Lin F, Zhao CY, Xu SH, Ma DY, Xiao ZZ, Xiao YS, Xu CA, Liu QH, Li J: **Germline-specific and sexually dimorphic expression of a dead end gene homologue in turbot (Scophthalmus maximus)**. Theriogenology 2013, **80**(6):665-672.

218. Aramaki S, Kubota K, Soh T, Yamauchi N, Hattori MA: **Chicken dead end homologue protein is a nucleoprotein of germ cells including primordial germ cells**. The Journal of Reproduction and Development 2009, **55**(2):214-218.

219. Wang D, Manali D, Wang T, Bhat N, Hong N, Li Z, Wang L, Yan Y, Liu R, Hong Y: **Identification of pluripotency genes in the fish medaka**. International Journal of Biological Sciences 2011, **7**(4):440-451.

220. Yang M, Deng B, Geng L, Li L, Wu X: **Pluripotency factor NANOG promotes germ cell maintenance in vitro without triggering dedifferentiation of spermatogonial stem cells**. Theriogenology 2020, **148**:68-75.

221. Yoshida S, Sukeno M, Nabeshima Y-i: **A vasculature-associated niche for undifferentiated spermatogonia in the mouse testis**. Science 2007, **317**(5845):1722-1726.

222. Zhang Y, Su H, Luo F, Wu S, Liu L, Liu T, Yu B, Wu Y: **E-cadherin can be expressed by a small population of rat undifferentiated spermatogonia in vivo and in vitro**. In Vitro Cellular & Developmental Biology - Animal 2011, **47**(8):593-600.

223. Yang Q-E, Kim D, Kaucher A, Oatley MJ, Oatley JM: **CXCL12-CXCR4 signaling is required for the maintenance of mouse spermatogonial stem cells**. Journal of Cell Science 2013, **126**(Pt 4):1009-1020.

224. Melo LH, Melo RMC, Luz RK, Bazzoli N, Rizzo E: **Expression of Vasa, Nanos2 and Sox9 during initial testicular development in Nile tilapia (Oreochromis niloticus) submitted to sex reversal**. Reproduction, Fertility and Development 2019, **31**(10):1637-1646.

225. Kodama M, Yoshida M, Endo M, Kobayashi T, Oike A, Yasumasu S, Nakamura M: **Nanos3 of the frog Rana rugosa: Molecular cloning and characterization**. Development, Growth & Differentiation 2018, **60**(2):112-120.

226. Li M-Q, Luo A-L, Zhao P-W, Li T-T, Geng S-S, Liang X-W, Xu H-Y, Lu Y-Q, Lu S-S, Yang X-G et al: **Nanos2 is a molecular marker of inchoate buffalo spermatogonia**. Animal Reproduction Science 2017, **186**:44-51.

227. Giassetti MI, Goissis MD, Moreira PV, de Barros FRO, Assumpção MEODÁ, Visintin JA: **Effect of age on expression of spermatogonial markers in bovine testis and isolated cells**. Animal Reproduction Science 2016, **170**:68-74.

228. Herrid M, Davey RJ, Hill JR: **Characterization of germ cells from pre-pubertal bull calves in preparation for germ cell transplantation**. Cell and Tissue Research 2007, **330**(2):321-329.

229. Rathi R, Honaramooz A, Zeng W, Schlatt S, Dobrinski I: **Germ cell fate and seminiferous tubule development in bovine testis xenografts**. Reproduction 2005, **130**(6):923-929.

230. Kim KJ, Cho CM, Kim BG, Lee YA, Kim BJ, Kim YH, Kim CG, Schmidt JA, Ryu BY: **Lentiviral modification of enriched populations of bovine male gonocytes**. Journal of Animal Science 2014, **92**(1):106-118.

231. Silva AF, Escada-Rebelo S, Amaral S, Tavares RS, Schlatt S, Ramalho-Santos J, Mota PC: **Can we induce spermatogenesis in the domestic cat using an in vitro tissue culture approach?** PLoS One 2018, **13**(2):e0191912.

232. Escada-Rebelo S, Silva AF, Amaral S, Tavares RS, Paiva C, Schlatt S, Ramalho-Santos J, Mota PC: **Spermatogonial stem cell organization in felid testis as revealed by Dolichos biflorus lectin**. Andrology 2016, **4**(6):1159-1168.

233. Lee KH, Lee R, Lee WY, Kim DH, Chung HJ, Kim JH, Kim NH, Choi SH, Kim JH, Song H: **Identification and in vitro derivation of spermatogonia in beagle testis**. PLoS One 2014, **9**(10):e109963.

234. Lee W-Y, Park H-J, Lee R, Lee J-H, Jhun H, Hur T-Y, Song H: **Analysis of putative biomarkers of undifferentiated spermatogonia in dog testis**. Animal Reproduction Science 2017, **185**:174-180.

235. Hur T-Y, Lee S-H, Ock S-A, Song H, Park H-J, Lee R, Sung S-H, Jhun H, Lee W-Y: **Dose-dependent effects of busulfan on dog testes in preparation for spermatogonial stem cell transplantation**. Laboratory Animal Research 2017, **33**(3):264-269.

236. Lee KH, Lee WY, Kim DH, Lee SH, Do JT, Park C, Kim JH, Choi YS, Song H: **Vitrified canine testicular cells allow the formation of spermatogonial stem cells and seminiferous tubules following their xenotransplantation into nude mice**. Scientific Reports 2016, **6**(1):21919.

237. Dong L, Kristensen SG, Hildorf S, Gul M, Clasen-Linde E, Fedder J, Hoffmann ER, Cortes D, Thorup J, Andersen CY: **Propagation of Spermatogonial Stem Cell-Like Cells From Infant Boys**. Frontiers in Physiology 2019, **10**(1155).

238. Kon Y, Endoh D, Iwanaga T: **Expression of protein gene product 9.5, a neuronal ubiquitin C-terminal hydrolase, and its developing change in Sertoli cells of mouse testis**. Molecular Reproduction and Development 1999, **54**(4):333-341.

239. Lee W-Y, Do JT, Park C, Kim JH, Chung H-J, Kim K-W, Gil C-H, Kim N-H, Song H: **Identification of putative biomarkers for the early stage of porcine spermatogonial stem cells using next-generation sequencing**. PLoS One 2016, **11**(1):e0147298.

240. Park H-J, Lee W-Y, Park C, Hong K-H, Kim J-H, Song H: **Species-specific expression of phosphoglycerate kinase 2 (PGK2) in the developing porcine testis**. Theriogenology 2018, **110**:158-167.

241. Park HJ, Lee W-Y, Kim JH, Park C, Song H: **Expression patterns and role of SDF-1/CXCR4 axis in boar spermatogonial stem cells**. Theriogenology 2018, **113**:221-228.

242. Lee W-Y, Lee K-H, Heo Y-T, Kim N-H, Kim J-H, Kim J-H, Moon S-H, Chung H-J, Yoon M-J, Song H: **Transcriptional coactivator undifferentiated embryonic cell transcription factor 1 expressed in spermatogonial stem cells: A putative marker of boar spermatogonia**. Animal Reproduction Science 2014, **150**(3):115-124.

243. Lee W-Y, Park H-J, Lee R, Lee K-H, Kim Y-H, Ryu B-Y, Kim N-H, Kim J-H, Kim J-H, Moon S-H et al: **Establishment and in vitro culture of porcine spermatogonial germ cells in low temperature culture conditions**. Stem Cell Research 2013, **11**(3):1234-1249.

244. Luo J, Rodriguez-Sosa JR, Tang L, Bondareva A, Megee S, Dobrinski I: **Expression pattern of acetylated alpha-tubulin in porcine spermatogonia**. Molecular Reproduction and Development 2010, **77**(4):348-352.

245. Lee R, Lee W-Y, Park H-J, Ha W-T, Woo J-S, Chung H-J, Lee J-H, Hong K, Song H: **Stage-specific expression of DDX4 and c-kit at different developmental stages of the porcine testis**. Animal Reproduction Science 2018, **190**:18-26.

246. Park H-J, Lee W-Y, Park C, Hong K, Song H: **CD14 is a unique membrane marker of porcine spermatogonial stem cells, regulating their differentiation**. Scientific Reports 2019, **9**(1):9980.

247. Honaramooz A, Megee SO, Rathi R, Dobrinski I: **Building a testis: formation of functional testis tissue after transplantation of isolated porcine (Sus scrofa) testis cells**. Biology of Reproduction 2007, **76**(1):43-47.

248. Zhao H, Li T, Yang H, Mehmood MU, Lu Y, Liang X, Yang X, Xu H, Lu K, Lu S: **The effects of growth factors on proliferation of spermatogonial stem cells from Guangxi Bama mini-pig**. Reproduction in Domestic Animals 2019, **54**(12):1574-1582.

249. Webster D, Bondareva A, Solin S, Goldsmith T, Su L, Lara NdLeM, Carlson DF, Dobrinski I: **Targeted Gene Editing in Porcine Spermatogonia**. Frontiers in Genetics 2021, **11**(1861).

250. Olejnik J, Suchowerska N, Herrid M, Jackson A, Jackson M, Andronicos NM, Hinch GN, Hill JR: **Sensitivity of spermatogonia to irradiation varies with age in pre-pubertal ram lambs**. Animal Reproduction Science 2018, **193**:58-67.

251. Arregui L, Rathi R, Megee SO, Honaramooz A, Gomendio M, Roldan ER, Dobrinski I: **Xenografting of sheep testis tissue and isolated cells as a model for preservation of genetic material from endangered ungulates**. Reproduction 2008, **136**(1):85-93.

252. Deng S, Wang X, Wang Z, Chen S, Wang Y, Hao X, Sun T, Zhang Y, Lian Z, Liu Y: **In vitro production of functional haploid sperm cells from male germ cells of Saanen dairy goat**. Theriogenology 2017, **90**:120-128.

253. Heidari B, Gifani M, Shirazi A, Zarnani A-H, Baradaran B, Naderi MM, Behzadi B, Borjian-Boroujeni S, Sarvari A, Lakpour N et al: **Enrichment of undifferentiated type a spermatogonia from goat testis using discontinuous percoll density gradient and differential plating**. Avicenna Journal of Medical Biotechnology 2014, **6**(2):94-103.

254. Medrano JV, Rombaut C, Simon C, Pellicer A, Goossens E: **Human spermatogonial stem cells display limited proliferation in vitro under mouse spermatogonial stem cell culture conditions**. Fertility and Sterility 2016, **106**(6):1539-1549.e1538.

255. Kim B-G, Cho CM, Lee Y-A, Kim B-J, Kim K-J, Kim Y-H, Min K-S, Kim CG, Ryu B-Y: **Enrichment of testicular gonocytes and genetic modification using lentiviral transduction in pigs**. Biology of Reproduction 2010, **82**(6):1162-1169.

256. Eildermann K, Aeckerle N, Debowski K, Godmann M, Christiansen H, Heistermann M, Schweyer S, Bergmann M, Kliesch S, Gromoll J et al: **Developmental expression of the pluripotency factor Sal-like protein 4 in the monkey, human and mouse testis: restriction to premeiotic germ cells**. Cells Tissues Organs 2012, **196**(3):206-220.

257. Hobbs Robin M, Fagoonee S, Papa A, Webster K, Altruda F, Nishinakamura R, Chai L, Pandolfi Pier P: **Functional antagonism between Sall4 and Plzf defines germline progenitors**. Cell Stem Cell 2012, **10**(3):284-298.

258. Park H-J, Lee R, Lee W-Y, Kim J-H, Do JT, Park C, Song H: **Stage-specific expression of Sal-like protein 4 in boar testicular germ cells**. Theriogenology 2017, **101**:44-52.

259. Izadyar F, Spierenberg G, Creemers L, Ouden Kd, Rooij Dd: **Isolation and purification of type A spermatogonia from the bovine testis**. Reproduction 2002, **124**(1):85.

260. Ertl C, Wrobel KH: **Distribution of sugar residues in the bovine testis during postnatal ontogenesis demonstrated with lectin-horseradish peroxidase conjugates**. Histochemistry 1992, **97**(2):161-171.

261. Almunia J, Nakamura K, Murakami M, Takashima S, Takasu M: **Characterization of domestic pig spermatogenesis using spermatogonial stem cell markers in the early months of life**. Theriogenology 2018, **107**:154-161.

262. Valdivia M, Castaneda-Zegarra S, Levano G, Lazo J, Reyes J, Bravo Z, Santiani A, Mujica F, Ruiz J, Gonzales GF: **Spermatogonial stem cells identified by molecular expression of PLZF, integrin beta1 and reactivity to Dolichos biflorus agglutinin in alpaca adult testes**. Andrologia 2019, **51**(6):e13283.

263. Valdivia M, Reyes J, Bravo Z, Cancho C, Castaneda S, Limaymanta O, Woll P, Santiani A, Gonzales GF: **In vitro culture of spermatogonial stem cells isolated from adult alpaca (Vicugna pacos) testes analysed with Dolichos biflorus by flow cytometry**. Andrologia 2019, **51**(6):e13269.

264. Aponte PM, Soda T, van de Kant HJG, de Rooij DG: **Basic features of bovine spermatogonial culture and effects of glial cell line-derived neurotrophic factor**. Theriogenology 2006, **65**(9):1828-1847.

265. Awang-Junaidi AH, Honaramooz A: **Optimization of culture conditions for short-term maintenance, proliferation, and colony formation of porcine gonocytes**. Journal of Animal Science and Biotechnology 2018, **9**(1):8.

266. Goissis MD, Giassetti MI, Worst RA, Mendes CM, Moreira PV, Assumpção MEOA, Visintin JA: **Spermatogonial stem cell potential of CXCR4-positive cells from prepubertal bull testes**. Animal Reproduction Science 2018, **196**:219-229.

267. Lee G, Jung H, Yoon M: **The Lin28 expression in stallion testes**. PLoS One 2016, **11**(10):e0165011.

268. Rode K, Weider K, Damm OS, Wistuba J, Langeheine M, Brehm R: **Loss of connexin 43 in Sertoli cells provokes postnatal spermatogonial arrest, reduced germ cell numbers and impaired spermatogenesis**. Reproductive Biology 2018, **18**(4):456-466.

269. Jung H, Roser JF, Yoon M: **UTF1, a Putative Marker for Spermatogonial Stem Cells in Stallions**. PLoS One 2014, **9**(10):e108825.

270. Sharma S, Schlatt S, Van Pelt A, Neuhaus N: **Characterization and population dynamics of germ cells in adult macaque testicular cultures**. PLoS One 2019, **14**(6):e0218194.

271. Kanatsu-Shinohara M, Toyokuni S, Shinohara T: **CD9 is a surface marker on mouse and rat male germline stem cells**. Biol Reprod 2004, **70**(1):70-75.

272. Van Saen D, Goossens E, Bourgain C, Ferster A, Tournaye H: **Meiotic activity in orthotopic xenografts derived from human postpubertal testicular tissue**. Human Reproduction 2010, **26**(2):282-293.

273. Poels J, Abou-Ghannam G, Herman S, Van Langendonckt A, Wese F-X, Wyns C: **In search of better spermatogonial preservation by supplementation of cryopreserved human immature testicular tissue xenografts with N-acetylcysteine and testosterone**. Frontiers in Surgery 2014, **1**:47-47.

274. Heckmann L, Langenstroth-Röwer D, Pock T, Wistuba J, Stukenborg JB, Zitzmann M, Kliesch S, Schlatt S, Neuhaus N: **A diagnostic germ cell score for immature testicular tissue at risk of germ cell loss**. Human Reproduction 2018, **33**(4):636-645.

275. Sharma S, Sandhowe-Klaverkamp R, Schlatt S: **Differentiation of Testis Xenografts in the Prepubertal Marmoset Depends on the Sex and Status of the Mouse Host**. Frontiers in Endocrinology 2018, **9**(467).

276. Chan F, Oatley MJ, Kaucher AV, Yang Q-E, Bieberich CJ, Shashikant CS, Oatley JM: **Functional and molecular features of the Id4+ germline stem cell population in mouse testes**. Genes & Development 2014, **28**(12):1351-1362.

277. Ferguson L, How JJ, Agoulnik AI: **The fate of spermatogonial stem cells in the cryptorchid testes of RXFP2 deficient mice**. PLoS One 2013, **8**(10):e77351.

278. Aloisio GM, Nakada Y, Saatcioglu HD, Peña CG, Baker MD, Tarnawa ED, Mukherjee J, Manjunath H, Bugde A, Sengupta AL et al: **PAX7 expression defines germline stem cells in the adult testis**. The Journal of Clinical Investigation 2014, **124**(9):3929-3944.

279. Park H-J, Lee WY, Lee R, Park J-k, Hong K-H, Park C, Song H: **Expression of paired box protein PAX7 in prepubertal boar testicular gonocytes**. Acta Histochemica 2020, **122**(6):151595.

280. Sharma A, Shah SM, Saini N, Mehta P, Kumar BSB, Dua D, Singh MK, Singla SK, Palta P, Manik RS et al: **Optimization of serum-free culture conditions for propagation of putative buffalo (Bubalus bubalis) spermatogonial stem cells**. Cellular Reprogramming 2019, **21**(1):1-10.

281. Lee YA, Kim YH, Ha SJ, Kim KJ, Kim BJ, Kim BG, Choi SH, Kim IC, Schmidt JA, Ryu BY: **Cryopreservation of porcine spermatogonial stem cells by slow-freezing testis tissue in trehalose**. Journal of Animal Science 2014, **92**(3):984-995.
